# Virome and Microbiome of Florida Bats Illuminate Viral Co-Infections, Dietary Viral Signals, and Gut Microbiome Shifts

**DOI:** 10.3390/microorganisms13112625

**Published:** 2025-11-19

**Authors:** Julia E. Paoli, Thanaporn Thongthum, Maclean Bassett, Jakob Beardsley, Massimiliano S. Tagliamonte, Melanie N. Cash, Jason Spertus Newman, Lisa M. Smith, Benjamin D. Anderson, Marco Salemi, Kuttichantran Subramaniam, Michael E. von Fricken, Elizabeth Braun de Torrez, Verity Mathis, Carla N. Mavian

**Affiliations:** 1 Emerging Pathogens Institute, University of Florida, Gainesville, FL 32610, USAm.bassett@ufl.edu (M.B.); mcash@pathology.ufl.edu (M.N.C.);; 2 Department of Environmental and Global Health, College of Public Health and Health Professions, University of Florida, Gainesville, FL 32610, USA; 3 Department of Pathology, Immunology and Laboratory Medicine, College of Medicine, University of Florida, Gainesville, FL 32610, USA; 4 One Health Center of Excellence, College of Public Health and Health Professions, University of Florida, Gainesville, FL 32610, USA; 5 Bioinformatics Core, Interdisciplinary Center for Biotechnology Research, University of Florida, Gainesville, FL 32610, USA; 6 Terrestrial Mammal Research, Florida Fish and Wildlife Research Institute, Florida Fish and Wildlife Conservation Commission, Gainesville, FL 32601, USA; 7 Department of Infectious Diseases and Immunology, College of Veterinary Medicine, University of Florida, Gainesville, FL 32610, USA; 8 Global Health Program Smithsonian’s, National Zoo & Conservation Biology, Washington, DC 20008, USA; 9 Florida Museum of Natural History, University of Florida, Gainesville, FL 32611, USA; vmathis@flmnh.ufl.edu

**Keywords:** alphacoronavirus, astrovirus, Brazilian free-tailed bats, southeastern myotis, Florida, museomics, metagenomics

## Abstract

Florida’s bat virome remains poorly characterized despite the state’s high bat species diversity and conservation importance. We characterized viral metagenomes from rectal tissues, anal swabs, and feces of *Myotis austroriparius* and *Tadarida brasiliensis* sampled across north Florida. We recovered a near-complete Hubei virga-like virus 2 (HVLV2) genome from *T. brasiliensis* feces, a finding consistent with an arthropod-derived dietary signal rather than active bat infection. An *Alphacoronavirus* (AlphaCoV) was detected in two *M. austroriparius* specimens, including one with a putative co-infection involving an *Astrovirus* (AstV), the first detection of AstV in Florida bats to date. Parallel profiling of the *M. austroriparius* gut microbiome highlighted compositional differences in the co-infected individual relative to AlphaCoV-only and virus-negative bats, suggestive of potential associations between viral detection and gut microbial shifts. Our study expands the known viral diversity in Florida bat populations, and demonstrates how metagenomics can simultaneously illuminate host diet, viral exposure, and gut microbial ecology. This approach provides a scalable framework for monitoring how diet, microbiome composition, and environmental pressures shape the bat virome, and inform conservation and zoonotic risk assessments.

## 1. Introduction

Bats (order *Chiroptera*) are known natural reservoirs for several emerging viruses with pandemic potential including filoviruses, paramyxoviruses, and coronaviruses [1]. Compared to other mammals, bats host the greatest number of zoonotic pathogens owing in part to their remarkable tolerance for viral infection [2]. Since the occurrence of several high-profile outbreaks linked to spillover from bats, research has primarily focused on investigating the virome composition of bats as a surveillance tool [3,4,5,6,7,8]. The bat gut microbiome may play a key role in regulating host immunity and antiviral responses, potentially contributing to bats’ increased tolerance to viral infections and their role as reservoirs for zoonotic pathogens [9,10,11]. However, the mechanisms underlying these interactions remain poorly understood [11,12,13]. Further studies are needed to determine how the gut microbiome of bats may be affecting pathogen exposure, evolution of tolerance, and spillover mechanisms [11,14].

Bats play a crucial role in maintaining healthy ecosystems and supporting agricultural production by controlling insect populations through their insectivorous diets [15,16]. In the United States, bats frequently consume agricultural pests responsible for significant economic losses, providing natural pest control services valued at billions of dollars in annual crop savings [15,17]. Bat feeding habits have important effects on the composition and function of their gut microbiomes, with insectivorous bats found to have greater microbial community diversity than frugivorous and hematophagous species [10,18,19]. In addition to diet, the gut microbial composition is influenced by host factors such as species, age, and sex, as well as environmental variables including season and geographic location [18,20,21,22,23,24,25].

The use of metagenomic next-generation sequencing (mNGS) has greatly expanded knowledge of the diversity of bat-borne viruses worldwide [5,7,26,27,28,29]. The agnostic nature of mNGS allows for detection of diet-associated viruses from insects, such as members of the *Dicistroviridae* and *Iflaviridae* families, in addition to mammalian viruses [30,31]. Previous research has emphasized the need to characterize viral diversity among North American bat species, as most studies of bat-borne viruses have focused on Asia, Europe, and Africa [32,33,34]. In North America, several bat-borne alphacoronaviruses (AlphaCoVs) have been identified in at least six species of bats, including those from Brazilian free-tailed bats (*Tadarida brasiliensis*) in Florida and other states [32,35]. Astroviruses (AstVs), which belong to the genus *Mamastrovirus* in mammals and *Avastrovirus* in birds, infect a wide range of hosts, including humans, and generally exhibit low virus–host specificity [36]. Despite their zoonotic relevance, only one complete AstV genome from North American bats is publicly available; it was sequenced from bat feces collected in a mixed-species bat roost in California [37].

Florida harbors thirteen native species of bats year-round, including the Southeastern myotis *(Myotis austroriparius*) and the Brazilian free-tailed bat (*T. brasiliensis*) [38]. Both species are insectivorous and presumed to be largely confined within the state, as the warm climate and consistent food availability reduce the need for long-distance migrations [38]. Despite this diversity of bat species, the virome, microbiome and diet of Florida bats remain poorly studied. Published work has only documented AlphaCoV [35], with no AstV found in Florida bat populations to date. Similarly, few studies have used DNA metabarcoding to characterize the diet of Florida bats, though the research that has been conducted highlights their ecological and economic importance, particularly through consumption of agricultural pests [39,40,41]. Research on the microbiome of Florida’s bats is limited, and to our knowledge, no published studies have examined the microbiome of the state’s bat population.

To better understand the viruses circulating among two of Florida’s common native bat species, we used mNGS to examine the virome of *M. austroriparius* and *T. brasiliensis* sampled across north Florida. We detected one *M. austroriparius* specimen harboring a potential co-infection of AstV and AlphaCoV, as well as another *M. austroriparius* specimen with only AlphaCoV. In addition, we recovered a near-full-length HVLV2 genome from *T. brasiliensis* feces, consistent with dietary intake of mosquitoes. Finally, we characterized the composition and diversity of the gut microbiota in the *M. austroriparius* museum specimens to explore potential associations between viral presence and microbial composition shifts. Our findings provide insight on the understudied virome of native Florida bats, which will aid bat-conservation efforts and public health preparedness.

## 2. Materials and Methods

### 2.1. Sample Preparation from Whole Frozen Bat Specimens

**Figure 1:**
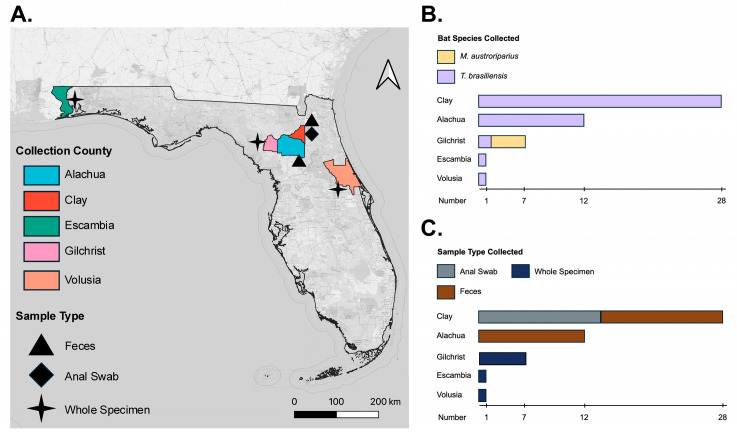
Map of sampling across Florida counties. The map indicates sampling sites where either whole specimens or fecal samples were collected: (**A**) map of the state of Florida with counties highlighted where bat samples were collected and the sample type collected by county; (**B**) distribution of bat species sampled by county; (**C**) distribution of sample type collected by county.

We obtained adult, whole specimens of *T. brasiliensis* (N = 5) and *M. austroriparius* (N = 5) from the Florida Museum of Natural History (FLMNH) (Table 1, Figure 1). These bats belonged to a mixed-species roost under the roof of a fire station in Gilchrist County, Florida during December 2021. The specimens were donated to the FLMNH after bats were killed accidentally during a power washing event. Following death, a wildlife rehab center brought the frozen carcasses to the FLMNH where they were weighed, sexed, and stored at −20 °C for preservation (Table S1). From the FLMNH archives, we additionally obtained frozen *T. brasiliensis* adult whole specimens collected from Escambia (N = 1) and Volusia (N = 1) counties, and one specimen from an unknown Florida county (Table 1, Figure 1). These three specimens were given to FLMNH from wildlife rehab centers, and the causes of death were unknown at the time of donation. The whole animal specimen was dissected, and the rectum was removed from each bat using a sterile scalpel (Thermo Fisher Scientific, Waltham, MA, USA). The rectum was then transferred into a sterile Petri dish, and another sterile scalpel was used to excise appropriately sized fragments (10 mm diameter) for RNA extraction. Excised rectal tissues were placed in RNAlater (Thermo Fisher Scientific, Waltham, MA, USA), then homogenized using a motorized pestle (Thermo Fisher Scientific, Waltham, MA, USA) and used immediately for RNA extraction. The samples were donated to the FLMNH and were not collected for a university-based project. Therefore, an Institutional Animal Care and Use Committee (IACUC) protocol was not required by the University of Florida.

### 2.2. Anal Swab and Fecal Sample Collection

We obtained opportunistic samples from adult *T. brasiliensis* that were actively and passively sampled on 21 November 2022, at one occupied roost (bat house) at Camp Blanding Joint Training Center in Clay County, Florida (Table 1, Figure 1). Fecal and anal swab samples were actively collected by capturing *T. brasiliensis* emerging from the roost using a 2 m × 2 m harp trap (Bat Conservation and Management, Carlisle, PA, USA). The harp trap was placed 1 m in front of the roost entrance one hour before sunset. Captured bats were immediately removed from the harp trap and placed individually in clean cloth bags for up to one hour or until they defecated, whichever occurred first. The sex, age, reproductive condition, mass, and forearm length for each bat were recorded. Feces were collected from the bag of each bat when present using ethanol flame-sterilized forceps. Anal swab samples were collected from each bat by first dipping a small sterile swab (VWR International, Radnor, PA, USA) in DNA/RNA Shield (Zymo Research, Irvine, CA, USA), then gently inserting the swab into the rectum and rotating 3–5 times. Bats were released at the site of capture immediately after processing. Fecal samples were passively collected at the same bat house by placing four 8 L buckets directly beneath the bat house. Approximately 1 h after the bats emerged, the buckets were removed and sterile forceps used to collect individual fecal pellets. Swabs and feces were immediately placed into 2 mL screw-cap microtubes (Sarstedt, Nümbrecht, Germany) containing DNA/RNA Shield (Zymo Research, Irvine, CA, USA) and stored at −80° C until analyzed. All live-capture and handling activities followed American Society of Mammalogists (ASM) guidelines [42] and were completed by experienced bat biologists with the Florida Fish and Wildlife Conservation Commission (FWC) under safe and humane protocols approved by the FWC.

In addition, on 10 April 2024, we obtained guano pellets (N = 12) from a *T. brasiliensis* colony roosting at the University of Florida’s bat houses in Alachua County using a passive sampling approach that did not require handling of bats (Table 1, Figure 1). Collection buckets were placed beneath the roosts to allow feces to accumulate naturally one hour prior to sunset to avoid interference with bat emergence from the house. After approximately 20 min, the buckets were retrieved, and individual guano pellets were collected using sterile forceps. Samples were pooled together for preservation in DNA/RNA Shield (Zymo Research, Irvine, CA, USA) and stored at −80 °C until processing. The fecal samples were collected non-invasively, without animal handling, manipulation, or roost disturbance; therefore, no IACUC protocol was required by the University of Florida.

### 2.3. RNA Extraction and NGS Library Preparation of Museum Samples and Anal Swabs

RNA was extracted from the museum specimen rectum tissue homogenate using the AllPrep DNA/RNA Mini Kit (Qiagen, Hilden, Germany) following the manufacturer’s protocol for total RNA isolation from animal tissues. For the anal swab samples, the ZymoBIOMICS DNA/RNA Miniprep kit was used following manufacturer’s protocol for RNA extraction (Zymo Research, Irvine, CA, USA). Subsequently, for both the museum tissue samples and anal swab samples, eluted RNA was treated with ezDNase (Thermo Fisher Scientific, Waltham, MA, USA) to remove residual genomic DNA, following the manufacturer’s protocol. First-strand cDNA was synthesized using SuperScript IV Reverse Transcriptase (Thermo Fisher Scientific, Waltham, MA, USA). Second-strand cDNA was synthesized using the Second Strand cDNA Synthesis Kit (Thermo Fisher Scientific, Waltham, MA, USA). The resulting double-stranded cDNA was purified using the Monarch PCR DNA Cleanup Kit (New England Biolabs, Ipswich, MA, USA).

Next-generation sequencing (NGS) libraries were prepared using the NEBNext Ultra II FS DNA Library Prep Kit for Illumina (New England Biolabs, Ipswich, MA, USA), following the low-input protocol (≤100 ng) with NEBNext Multiplex Oligos (Dual Index Primers Set 1) for Illumina (New England Biolabs, Ipswich, MA, USA). Libraries were amplified and bead-purified using AMPure XP beads (Beckman Coulter, Brea, CA, USA) at a 0.9× ratio. Library quality and fragment size distribution were assessed using the Agilent 4150 TapeStation system (Agilent Technologies, Santa Clara, CA, USA). Libraries containing primer-dimer peaks (<150 bp) were subjected to additional rounds of bead purification (0.9×) and re-analyzed until satisfactory quality was achieved. Final libraries were size-selected on the SageELF electrophoresis system (Sage Science, Beverly, MA, USA) to isolate fragments between 200 and 500 bp. Quantification of libraries was determined with the Qubit dsDNA HS Assay Kit (Thermo Fisher Scientific, Waltham, MA, USA) on the Qubit 4 Fluorometer (Thermo Fisher Scientific, Waltham, MA, USA). The final pooled library was sequenced on a NovaSeq 6000 system (Illumina, San Diego, CA, USA) using the NovaSeq 6000 S1 Reagent Kit v1.5 (paired-end, 2 × 150) at the University of Florida Interdisciplinary Center for Biotechnology Research (UF ICBR). Fastq files were deposited in SRA under BioProject accession PRJNA1305506.

### 2.4. RNA Extraction and NGS Library Preparation of Fecal Samples

For RNA extraction, guano pellets were thawed and homogenized in DNA/RNA Shield (Zymo Research, Irvine, CA, USA) using a motorized pestle. The homogenate was centrifuged at 2000 rpm for 5 min, and the resulting supernatant was used for RNA extraction with the MagMAX Viral RNA Isolation Kit (Thermo Fisher Scientific, Waltham, MA, USA). To remove potential PCR inhibitors, RNA samples were further purified using the OneStep PCR Inhibitor Removal Kit (Zymo Research, Irvine, CA, USA). RNA was extracted from guano pellets according to the previously published RAPIDprep assay [43] for the following steps: gDNA removal, rRNA depletion, first-strand cDNA synthesis, second-strand cDNA synthesis, and double-stranded cDNA cleanup. Two in-house modifications were made to the original RAPIDprep protocol to reduce reagent usage. First, the QIAseq FastSelect (Qiagen, Hilden, Germany) dilution mix was prepared at a 1:10 ratio by combining 1 µL of 5S/16S/23S rRNA removal reagent, 1 µL of HMR (human/mouse/rat) depletion reagent, and 8 µL of nuclease-free water. Second, the Sequenase dilution was modified to use 1 unit per reaction, corresponding to 0.08 µL of Sequenase Version 2.0 DNA Polymerase (Thermo Fisher Scientific, Waltham, MA, USA) mixed with 0.92 µL of nuclease-free water per reaction.

NGS libraries were prepared from the purified cDNA with the NEBNext Ultra II FS DNA Library Prep Kit for Illumina (New England Biolabs, Ipswich, MA, USA), following the low-input protocol (≤100 ng) with NEBNext Multiplex Oligos (Dual Index Primers Set 1) for Illumina (New England Biolabs, Ipswich, MA, USA). Libraries were amplified and bead-purified (0.9×) using Omega Bio-tek Mag-Bind Total Pure NGS beads (Omega Bio-tek, Norcross, GA, USA). Library fragment size distribution was assessed using the Agilent 4150 TapeStation system (Agilent Technologies, Santa Clara, CA, USA). Libraries containing primer-dimer peaks (<150 bp) were subjected to additional rounds of bead purification (0.9×) and re-analyzed until satisfactory quality was achieved. Quantification of libraries was determined with the Qubit dsDNA HS Assay Kit (Thermo Fisher Scientific, Waltham, MA, USA) on the Qubit 4 Fluorometer (Thermo Fisher Scientific, Waltham, MA, USA). Final libraries were normalized and pooled for sequencing with the NextSeq 1000 platform (Illumina, San Diego, CA, USA) using the NextSeq 1000/2000 P1 Reagents cartridge (paired-end, 2 × 150 bp). Fastq files were deposited in SRA under BioProject accession PRJNA1305506.

### 2.5. Metagenomic Data Analysis

Fastq files were trimmed with Trimmomatic v.0.39 [44] to remove adapters and poor quality regions. De novo assembly was performed with MEGAHIT v.1.2.9 [45] to generate contigs which were then assessed with NCBI’s VecScreen to remove potential vector contamination (https://www.ncbi.nlm.nih.gov/tools/vecscreen; accessed 17 October 2025). Cleaned contigs were compared to the BLAST nt/nr v2.17.0 [46] and InterPro v101.0 [47,48] databases for taxonomic identification and genome annotation. We obtained single, contiguous contigs for all samples with one exception (Table 2): for sample Ma_Frozen_3, two non-overlapping AlphaCoV contigs were recovered (k119_6311 and k119_670). Fastq files were deposited in SRA under BioProject accession PRJNA1305506. Viral contigs were submitted to GenBank with the following accessions: PX462113 (contig k119_6311); PX470470 (contig k119_670); PX470471 (contig k119_1119); PX482536 (contig k119_11258); PX462114 (contig k141_4344) (Table S2).

### 2.6. Recombination Analysis

For each virus of interest, representative complete genomes and their associated metadata were compiled from NCBI Virus to generate background datasets [49]. Multiple sequence alignments of these full-genome datasets were produced with MAFFT v.7.520 [50] and manually polished in AliView v1.28 [51]. In the AlphaCoV dataset, two *Tadarida brasiliensis* bat alphacoronavirus 2 reference sequences (OP700657.1 and OP715780.1) from the same study were included and hereafter referred to by the abbreviated virus name ACoV-2-Tb. Because only partial genomes were obtained in this study, recombination analysis was restricted to the full-genome background datasets, which were assessed using RDP5 with default settings for linear sequences [52]. Recombinant sequences were defined as those with significant evidence of recombination signal supported by at least six of the seven following methods: RDP, GENECONV, Chimaera, MaxChi, BootScan, SiScan, and 3Seq [52]. Identified recombinant regions were removed.

### 2.7. Phylogenetic Analysis

Following recombination analysis, we obtained several recombinant-free alignments for the publicly available genomes and produced several alignments with our viral contigs for phylogenetic analysis using MAFFT v.7.520 [50]. All alignments were constructed using single, contiguous de novo-assembled viral contigs, with one exception: to retain all AlphaCoV genomic signal from sample Ma_Frozen_3 in the phylogeny, a partial genome was constructed by inserting Ns between the two contigs (k119_6311 and k119_670), followed by masking these ambiguous regions during multiple sequence alignment in AliView v1.28 [51]. This resulted in a concatenated alignment consisting only of confidently assembled positions, for phylogenetic purposes only, referred to as ACoV-Ma3-FL-2021. The other recovered contigs used for phylogenetic analysis are hereafter referred to by the virus name followed by host specimen, location, and year as follows: (1) AstV-Ma4-FL-2021 (contig k119_11258) and (2) HVLV2-Tb15-FL-2024 (contig k141_4344). Maximum likelihood (ML) trees were obtained from (1) alignments based on the full contigs and (2) alignments based on individual genes: for AstV, the RNA-dependent RNA polymerase (RdRp) and open reading frame 2 (ORF2) genes; and for AlphaCoV, the spike, envelope, membrane, and nucleocapsid genes. ML phylogenetic tree reconstruction was performed with IQ-TREE v.2.2.2.7 [53] using the Bayesian information criteria (BIC) to infer the best nucleotide substitution model based on the data and 2000 ultrafast bootstrap replicates for support [54]. ML trees were outgroup rooted with closely related viruses and visualized in R using the ggtree v3.2.1 package [55]. Evidence of sufficiently robust phylogenetic signal of sequence data was assessed by likelihood mapping implemented in IQ-TREE v.2.2.2.7 per recommended guidelines: side/center areas of the likelihood mapping must include <40% of the unresolved quartets [56,57]. Pairwise genetic distance analysis was conducted with Molecular Evolutionary Genetics Analysis (MEGA) v.11 [58] using the models for the number of nucleotide differences and p-distance with the following parameters: Gamma distribution (shape parameter = 4.0), 100 bootstrap replicates for variance estimation, and gaps treated as complete deletions. The 1st, 2nd, 3rd, and non-coding sites were included.

### 2.8. DNA Extraction and 16S Library Preparation

From the *M. austroriparius* museum specimen rectum tissue homogenate, DNA was extracted using the AllPrep DNA/RNA Mini Kit (Qiagen, Hilden, Germany) following the manufacturer’s protocol for genomic DNA isolation from animal tissues. To assess the initial presence of bacterial DNA in the extracts, the V3–V4 hypervariable region of the bacterial 16S rRNA gene was amplified by PCR. Reactions were performed in a total volume of 25 μL using Q5 High-Fidelity 2X Master Mix (New England BioLabs, Ipswich, MA, USA). Each reaction contained 8 μL of input DNA, 12.5 μL of Q5 2X Master Mix, 2 μL of nuclease-free water, and 1.25 μL each of forward and reverse primers (10 μM): S-D-Bact-0341-b-S-17 (5′-CCTACGGGNGGCWGCAG-3′) and S-D-Bact-0785-a-A-21 (5′-GACTACHVGGGTATCTAATCC-3′) [59]. PCR cycling conditions were as follows: initial denaturation at 98 °C for 30 s; 35 cycles of 98 °C for 10 s, 55 °C for 30 s, and 72 °C for 30 s; followed by a final extension at 72 °C for 2 min. Amplicons were visualized by electrophoresis on a 1% agarose gel stained with ethidium bromide and compared against a 50–2000 bp DNA ladder (Bio-Rad, Hercules, CA, USA).

16S rRNA libraries were prepared using the Quick-16S Library Prep Kit (Zymo Research, Irvine, CA, USA) according to the manufacturer’s protocol for low microbial DNA samples. Library amplification was confirmed by nucleic acid quantification using the 4150 TapeStation System (Agilent Technologies, Santa Clara, CA, USA). Confirmed libraries were pooled according to the Quick-16S protocol and the final pooled library was quantified using both a Qubit fluorometer (Thermo Fisher Scientific, Waltham, MA, USA) and TapeStation analysis. Final libraries were diluted to 4 nM, then denatured and diluted to a final concentration of 10 pM with a 15% PhiX (Illumina, San Diego, CA, USA) spike-in. Libraries were sequenced on the MiSeq platform (Illumina, San Diego, CA, USA) using the MiSeq Reagent Kit v3 (paired-end, 2 × 300 bp). Fastq files were deposited in SRA under BioProject accession PRJNA1305506.

### 2.9. 16S rRNA Reads Processing and Statistical Analysis

Raw paired-end 16S rRNA gene sequences were processed using Quantitative Insights Into Microbial Ecology 2 (QIIME2) v2024.10 [60]. Sequences were quality filtered, joined, and denoised using the Deblur plugin [61] with a trim length of 400 base pairs, a mean per-nucleotide error threshold of 0.00125, and a minimum read count of 10 per sample. These parameters generated amplicon sequence variants (ASVs) and removed chimeric and low-abundance sequences. A rooted phylogenetic tree was constructed with the Fasttree pipeline [62] for downstream diversity analyses. For taxonomic assignment, a Naive Bayes classifier was trained on SILVA [63,64,65] 138 reference sequences trimmed in silico to the V3–V4 region using primers 341F (CCTACGGGNGGCWGCAG) and 806R (GACTACHVGGGTATCTAATCC) [65,66]. Core diversity metrics were computed using the core-metrics-phylogenetic pipeline with a rarefaction depth of 6000 sequences per sample, based on alpha rarefaction curves showing a plateau in observed feature richness. Alpha diversity was assessed using Shannon diversity index, implemented in QIIME2’s v2024.10 alpha-group-significance plugin. Beta diversity was evaluated using Bray–Curtis dissimilarity [67], weighted UniFrac [68], and unweighted UniFrac [69] distances. The results were visualized using principal coordinate analysis (PCoA). Differences in community composition across sample types were assessed using Kruskal–Wallis tests [70,71] and Permutational Multivariate Analysis of Variance (PERMANOVA) with 999 permutations [72]. We did not perform Kruskal–Wallis tests or PERMANOVA to compare bacterial composition across samples because each group comprised a single sample (no biological replicates).

### 2.10. Geospatial Mapping

Maps were generated with QGIS v.3.28.1 using GPS coordinates collected during sampling events (QGIS Association, 2024). Shapefiles for the state and counties of Florida were obtained from https://geodata.floridagio.gov/ (accessed 8 July 2025).

## 3. Results

### 3.1. First Detection of Astrovirus Detection in Florida Bats

We performed mNGS on RNA from rectal tissue dissected from five adult *M. austroriparius* and *T. brasiliensis* whole frozen specimens housed at FLMNH to assess viromes (Table 1, Figure 1). We did not detect any viral contigs (mammalian nor insect) in the five *T. brasiliensis* frozen museum specimens tested, nor in *M. austroriparius* specimens: Ma_Frozen_1, Ma_Frozen_2, and Ma_Frozen_5 (Table S1). In *M*. *austroriparius* specimen Ma_Frozen_4 we recovered a single *Astrovirus* (AstV) contig (k119_11258) that was 3292 bp long (accounting for 51% of the AstV genome) (Table 2). Hereafter, the contig will be referred to as AstV-Ma4-FL-2021 (Table S2). BLASTN v2.17.0 analysis showed the highest nucleotide identity to bat AstV from *Myotis daubentoniid* sampled in Denmark in 2015 (MZ218054.1; 80% nucleotide identity) and from *Myotis chinensis* sampled in China in 2020 (OR951016.1; 80% nucleotide identity). AstV-Ma4-FL-2021 spans 54% of the ORF1b and 100% of ORF2 plus the 3′ untranslated region (Figure S1); within ORF1b, it covers 69% of the RdRp gene (Figure S1).

According to current International Committee on Taxonomy of Viruses (ICTV) guidelines, the *Astroviridae* family comprises two genera: *Avastrovirus*, which infect birds, and *Mamastrovirus*, which infect mammals [73]. We therefore compiled a reference dataset including all *Mamastrovirus* reference sequences from GenBank, along with the sequences showing the highest nucleotide identity to AstV-Ma4-FL-2021 based on BLAST v2.17.0 analysis (Table S3). Recombination analysis indicated evidence of recombination in reference datasets for the full genome and ORF2; however, there was no evidence of recombination occurring in the RdRp gene. Using recombination free alignments, we calculated pairwise genetic distance which showed that AstV-Ma4-FL-2021 shares 70–71% nucleotide identity across the ORF1b-ORF2 region with three *Myotis*-associated bat AstVs (MZ218054.1, MZ218053.1, and OR951016.1) (Table S4), all currently unclassified within *Astroviridae*. These three genomes showed no evidence of recombination. Within RdRp, sequence identity was 72–73% (p-distance of 0.27–0.28; standard error = 0.02). Across ORF2, identity was 68% (p-distance of 0.33; standard error = 0.01), increasing to 81% (p-distance of 0.19; standard error = 0.01) within the conserved N-terminal region of the capsid protein.

**Figure 2:**
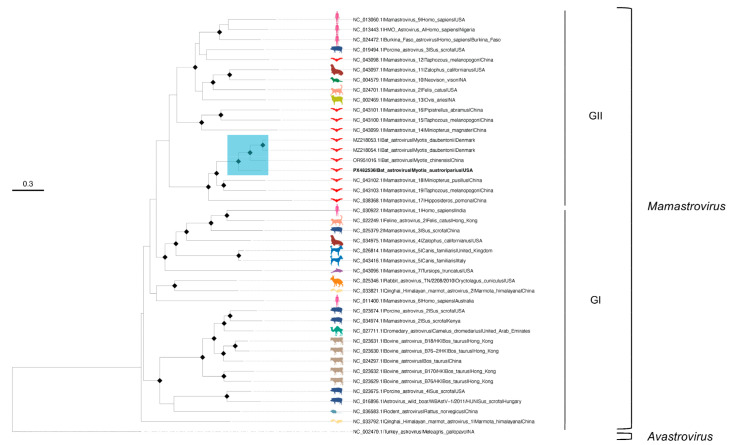
ML tree of AstV found in *M. austroriparius* sampled in Gilchrist County, FL in 2021 (specimen Ma_Frozen_4). The tree is based on the concatenated alignment of *Mamastrovirus* genomes with the study sample (GTR+F+R5). *Mamastrovirus* genogroups are labeled as GI and GII. The subclade containing AstV-Ma4-FL-2021 (GenBank PX482536), indicated in bold, is highlighted in blue. Sequence hosts are depicted and colored by host species, with bats highlighted in red. Diamonds at nodes indicate ultrafast bootstrap values above 90. Viruses labeled by GenBank accession number, name, and host. Scale bar in nucleotide substitutions per site. Outgroup rooted by *Avastrovirus* Turkey Astrovirus (NC_002480.1).

The ML phylogenies were inferred for AstV-Ma4-FL-2021 and individual gene (RdRp, ORF2) alignments, rooted with an avian outgroup (Turkey astrovirus; NC_002470.1). BIC selected GTR+F+R5 (AstV-Ma4-FL-2021), GTR+F+I+G4 (RdRp), and TVMe+R5 (ORF2). Likelihood mapping analysis indicated robust phylogenetic signal for all phylogenies (Figure S2). The full contig tree resolved two *Mamastrovirus* genogroups (I and II) [74,75] with all bat-derived sequences—including AstV-Ma4-FL-2021—clustering in genogroup II (Figure 2). Within this genogroup, AstV-Ma4-FL-2021 formed a well-supported monophyletic subclade composed exclusively of bat AstVs, grouping most closely with the unclassified *Myotis*-associated sequences (MZ218054.1, MZ218053.1, OR951016.1) and with *Mamastrovirus* 17 (NC_038368.1), *Mamastrovirus* 18 (NC_043102.1), and *Mamastrovirus* 19 (NC_043103.1). In gene-specific trees, AstV-Ma4-FL-2021 showed congruent placement within genogroup II (Figures S3 and S4); *Mamastrovirus* 17 clustered with the subclade in the ORF2 tree (Figure S3) but was excluded from the RdRp tree (Figure S4) due to insufficient coverage. Notably, in the RdRp tree the AstV-Ma4-FL-2021 subclade shared a more recent common ancestor with a neighboring clade (*Mamastrovirus* 14, 15, 16)—a relationship not recovered in the concatenated or ORF2 trees—potentially reflecting differing evolutionary constraints on the conserved RdRp versus the more variable capsid.

### 3.2. Co-Infection of an M. austroriparius with Astrovirus and a Bat Alphacoronavirus

In *M*. *austroriparius* specimen Ma_Frozen_4 we also detected an AlphaCoV contig (k119_1119) that was 1327 bp long. This finding suggests that Ma_Frozen_4 was co-infected with AlphaCoV and AstV (Table 2, Table S1). Contig k119_1119 shared 84% nucleotide identity with an unclassified AlphaCoV strain *Tadarida brasiliensis* bat alphacoronavirus 2 (ACoV-2-Tb; OP700657.1) (Table 2). Relative to OP700657.1, k119_1119 covered 51% of nucleocapsid gene and 100% of ORF7, ORF8, and the 3′ genomic region (Figure S5). From a second *M. austroriparius* specimen, Ma_Frozen_3, we recovered two additional AlphaCoV contigs: k119_6311 (925 bp; 81% identity to OP700657.1) and k119_670 (5309 bp; 81% identity to OP700657.1) (Table 2). Relative to OP700657.1, k119_6311 covered the 5′ genomic region and 5% of ORF1a, whereas k119_670 covered 42% of spike, 100% of ORF3, envelope, membrane, nucleocapsid, ORF7, ORF8, and the 3′ region (Table 2, Figure S5). For analytical purposes, a draft AlphaCoV genome was reconstructed by concatenating contigs k119_6311 and k119_670 (hereafter ACoV-Ma3-FL-2021) to facilitate phylogenetic placement. The AlphaCoV contig k119_1119 from sample Ma_Frozen_4 was short and identical to the corresponding region in k119_670 from sample Ma_Frozen_3; therefore, only contigs from Ma_Frozen_3 were used for downstream analyses.

To assess relatedness, we assembled a background dataset representing the 15 recognized AlphaCoV subgenera plus unclassified bat AlphaCoVs closely related to study contigs (Table S5). As expected for coronaviruses, recombination was detected in the reference dataset; recombinant regions were removed prior to analysis. No recombination was detected in ACoV-2-Tb, the closest lineage. Gene-level pairwise comparison of study contigs with reference genomes indicated that the genes for ORF1a, envelope, membrane, and nucleocapsid shared greatest identity with ACoV-2-Tb strains (Table S6). In the spike gene, our sample shared greatest identity (p-distance 0.29; standard error 0.01) with a *Myotis siligorensis* bat coronavirus sampled in China in 2019 (OQ175040.1) and was less similar to ACoV-2-Tb strains sampled from *T. brasiliensis* in Argentina in 2016–2017 (p-distance 0.36; standard error 0.01) (Table S6).

**Figure 3:**
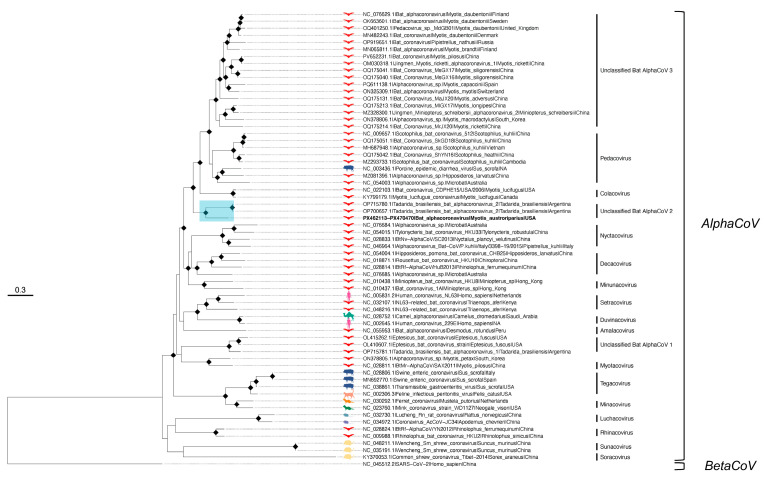
ML tree of AlphaCoV found in M. austroriparius sampled in Gilchrist County, FL in 2021 (specimen Ma_Frozen_3). The tree is based on concatenated alignment of ACoV-Ma3-FL-2021 (PX462113-PX470470) with alphacoronaviruses from all fifteen classified subgenera as well as unclassified sequences with close identity to ACoV-Ma3-FL-2021 (GTR+F+R6). The subclade containing ACoV-Ma3-FL-2021, indicated in bold, is highlighted in blue. Sequence hosts are depicted and colored by host species, with bats highlighted in red. Diamonds at nodes indicate ultrafast bootstrap values above 90. Viruses labeled by GenBank accession number, virus name, and host. Scale bar in nucleotide substitutions per site. Outgroup rooted by Betacoronavirus SARS-CoV-2 (NC_045512.2).

Based on the concatenated alignment, in the ML tree ACoV-Ma3-FL-2021 clustered in a well-supported subclade with ACoV-2-Tb strains from Argentina (OP700657.1, OP715780.1), sharing 81% nucleotide identity, respectively (Figure 3). ACoV-Ma3-FL-2021 occupied a basal, longer branch relative to the Argentine strains, consistent with a distinct lineage and/or unsampled diversity in Florida bat populations, geographic separation, different hosts and sampling times (Argentine sequences were sampled in 2016 and in 2017). A second putative species, named *Tadarida brasiliensis* bat alphacoronavirus 1 (ACoV-1-Tb; OP715781.1), forms a separate lineage that clusters with unclassified AlphaCoVs from *Eptesicus* bats collected in the USA and South Korea (here termed ‘Unclassified Bat AlphaCoV 1′) [76]. The most closely related classified subgenera to ACoV-Ma3-FL-2021 were *Pedacovirus* and *Colacovirus*, the latter currently comprising *Myotis lucifugus* coronaviruses collected in Colorado in 2006 (NC_022103.1) and in Canada in 2010 (KY799179.1) (Figure 3). Gene-specific phylogenies for envelope, membrane, and nucleocapsid recapitulated the concatenated-tree placement, with ACoV-Ma3-FL-2021 forming a well-supported subclade on a long branch basal to the Argentine ACoV-2-Tb lineage (Figure S6).

Phylogenetic analysis of the partial spike gene revealed the ACoV-2-Tb strains to not cluster with ACoV-Ma3-FL-2021 in a subclade and instead, they grouped in a distantly related, well-supported subclade with the *Decacovirus* Alphacoronavirus sp. WA3607 (NC_076685.1) (Figure S6). ACoV-Ma3-FL-2021 remained closely related to members of *Colacovirus*, *Pedacovirus*, as well as unclassified *Myotis*-associated coronaviruses in the spike phylogeny.

### 3.3. Detection of Hubei Virga-like Virus 2 from T. brasiliensis Feces

Although no viruses were detected in *T. brasiliensis* anal swabs or fecal samples from Clay County, we identified the unclassified RNA virus HVLV2 in fecal samples from *T. brasiliensis* roosting in Alachua County (Table 1, Figure 1). De novo assembly yielded a single viral contig of 9829 bp (k141_4344) with 99% nucleotide identity to publicly available HVLV2 sequences (Table 2). Contig k141_4344 spans 90% of the complete HVLV2 genome, including 99% of the polyprotein region; however none of the coat protein was recovered (Figure S7). Hereafter, contig k141_4344 will be referred to as HVLV2-Tb15-FL-2024.

**Figure 4:**
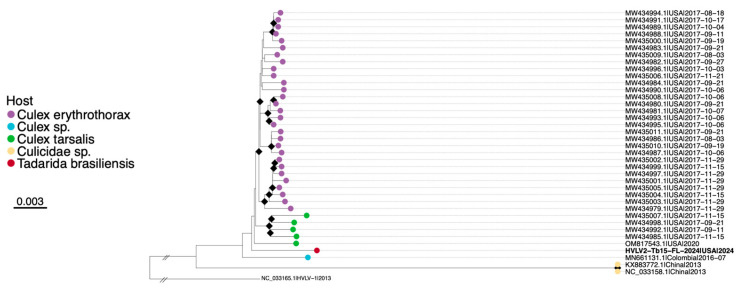
ML tree of HVLV2 found in *T. brasiliensis* guano sampled in Alachua County, FL in 2024 (specimen Tb_Feces_15). The tree is based on the concatenated alignment of the study sample with HVLV2 reference genomes (TVM+F+I). HVLV2-Tb15-FL-2024 (PX462114) is indicated in bold. Black diamonds at nodes indicate ultrafast bootstrap values above 90. Viruses labeled by GenBank accession number, country, and date. Tips are colored according to host species. Scale bar in nucleotide substitutions per site. Outgroup rooted by Hubei virga-like virus 1 (NC_033165.1) and truncated for visualization purposes.

A total of 37 HVLV2 genomes were retrieved from NCBI Virus predominately detected in mosquitoes of the *Culex* genus (Table S6). Recombination analysis showed no evidence of recombination between included sequences in the dataset. Pairwise distance analysis of the polyprotein revealed high nucleotide similarity between HVLV2-Tb15-FL-2024 and all included HVLV2 sequences (96–99.5%) (Table S7). The closest genomes to our sample were from *Culex erythrothorax* collected in California, USA, in 2017 (MW434980.1; MW434995.1; p-distance 0.005, SE 0.001) and *Culex tarsalis* sampled in the USA in 2020 and in California, USA, in 2017 (OM817543.1; MW434992.1; p-distance 0.006, SE 0.001). ML tree placed HVLV2-Tb15-FL-2024 within the clade of genomes obtained from *Culex* sp. mosquitoes, in between sequences from Colombia and U.S., although bootstrap support was low (Figure 4). HVLV2-Tb15-FL-2024 resides on a relatively long branch basal to U.S. sequences, consistent with geographic structure and/or unsampled diversity.

### 3.4. Microbiome Shifts in AlphaCoV and AstV Co-Infected Bat

We performed 16S rRNA gene sequencing on *M. austroriparius* samples and compared the gut microbiota of Ma_Frozen_3 (AlphaCoV positive) and Ma_Frozen_4 (AlphaCoV and AstV positive) with those of Ma_Frozen_1, Ma_Frozen_2, and Ma_Frozen_5 (virus-negative). For Ma_Frozen_4 and Ma_Frozen_5, we processed two preparation types (secondary-prepared and concentrated) and evaluated whether they could be combined for downstream analyses. Microbial community composition was highly similar between preparation types at both the genus and species levels (Figures S8 and S9). Shannon diversity did not differ (Kruskal–Wallis: H = 0.59, df = 1, *p* = 0.438; Figure S10). Beta diversity patterns based on Bray–Curtis dissimilarities showed minimal visual differences in community structure (Figure S11), and PERMANOVA detected no effect of preparation type (F = 0.1679, R^2^ = 0.077, *p* = 1.000; 23 permutations). Accordingly, we merged the two preparation types for each sample in subsequent analyses.

Visual presentation of alpha and beta diversity suggested alpha diversity (Shannon index) varied across specimens, with Ma_Frozen_4 exhibiting the highest diversity, followed by Ma_Frozen_2, Ma_Frozen_1, Ma_Frozen_3, and Ma_Frozen_5 (Figure S12). Beta diversity based on Bray–Curtis dissimilarity (PCoA) plot grouped Ma_Frozen_1 with Ma_Frozen_2 and Ma_Frozen_3 with Ma_Frozen_4, whereas Ma_Frozen_5 formed an outlier cluster (Figure S13). Weighted UniFrac PCoA, which incorporates phylogenetic relatedness and abundance, grouped Ma_Frozen_3 with Ma_Frozen_1/2 and resolved Ma_Frozen_4 separately; Ma_Frozen_5 again appeared as an outlier (Figure S14). Overall, these observations also indicated a possible trend toward higher alpha diversity and a phylogenetically distinct community in the AlphaCoV+AstV co-infected Ma_Frozen_4 relative to the AlphaCoV-only individual (Ma_Frozen_3) and virus-negative bats (Ma_Frozen_1/2); however, these patterns are purely descriptive and were not tested statistically.

**Figure 5:**
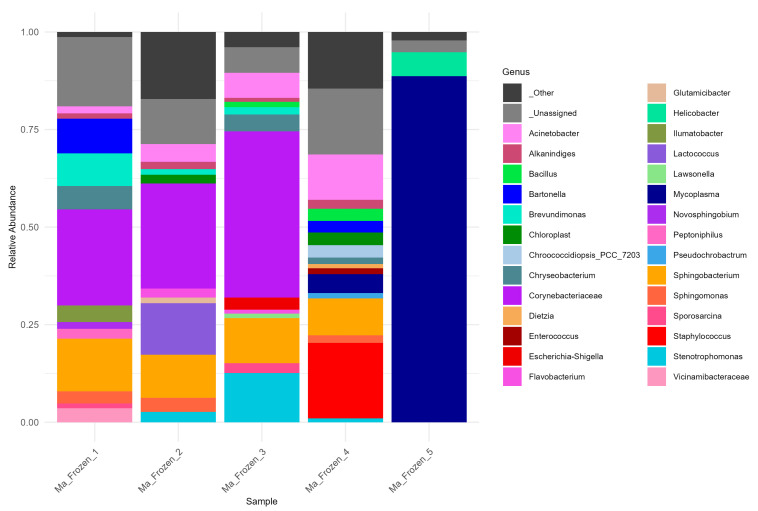
Genus-level bacterial composition of *M. austroriparius* frozen museum samples. Only taxa with ≥1% relative abundance are shown. For taxonomic assignment, a Naive Bayes classifier was trained on SILVA 138 reference sequences trimmed in silico to the V3–V4 region using primers 341F (CCTACGGGNGGCWGCAG) and 806R (GACTACHVGGGTATCTAATCC).

At the genus level, communities were compositionally distinct (Figure 5). The family *Corynebacteriaceae* (dominated by *Corynebacterium*) prevailed in Ma_Frozen_1/2/3 but was absent from Ma_Frozen_4, consistent with altered gut microbiota under co-infection. *Acinetobacter* and *Sphingobacterium* were prevalent across Ma_Frozen_1–4, with *Acinetobacter* relatively enriched in Ma_Frozen_3/4 and *Sphingobacterium* enriched in Ma_Frozen_1/2. In Ma_Frozen_4, no single genus dominated; notable taxa included *Mycoplasma*, *Acinetobacter*, *Sphingobacterium*, *Bartonella*, and *Staphylococcus.* By contrast, Ma_Frozen_5 was overwhelmingly dominated by *Mycoplasma* with minor *Helicobacter*. The *Bartonella* genus, present in Ma_Frozen_1 and Ma_Frozen_4, were taxonomically consistent with genuine Bartonella rather than typical mitochondrial (Rickettsiales) or chloroplast (Cyanobacteria/Chloroplast) origins, suggesting they are unlikely to represent organellar contamination. However, given the low-biomass and single-sample context, we cannot entirely exclude the possibility of *Alphaproteobacterial* background signals. At the species level, virus positive samples Ma_Frozen_3/4 showed higher abundance of *Acinetobacter iwoffi* compared to virus-negative samples (Figure S15).

## 4. Discussion

In this study, we analyzed the viromes and gut microbiomes of native north Florida bats. As the state of Florida experiences one of the highest human population growth rates in the United States, many bats in Florida are likely experiencing population declines due to loss of natural habitat from deforestation and human disturbance or destruction of roosting sites [77,78]. Bat habitat loss is strongly linked to poor bat health outcomes and increased human–bat interactions, which elevate the risk of zoonotic spillover [79,80]. We aimed to characterize the viral diversity circulating in two common bat species, *T. brasiliensis* and *M. austroriparius*, given the paucity of studies on Florida’s resident bat populations and their tendency to roost in urban areas, including in buildings, bridges, and culverts, making them particularly vulnerable to human interference [81,82,83,84]. We report here the first detection of AstV and AlphaCoV in *M. austroriparius* in Florida. Moreover, we found evidence of co-infection with both viruses in one individual. Such co-infections are plausible, as infection with one virus may weaken the host’s immune system and increase susceptibility to a secondary infection [85]. The AstV identified in this study clustered within *Mamastrovirus* genogroup II with *Myotis*-associated AstV collected in Denmark and in China [7,86]. As Florida *M. austroriparius* are not known to undertake long-distance migrations, cross-continental contact is unlikely; the relatively low nucleotide identity to available references more plausibly reflects undersampling of New World *Myotis* astroviruses and potential host-associated divergence. Collectively, these findings suggest AstV-Ma4-FL-2021 may represent a novel *Mamastrovirus* lineage associated with native *M. austroriparius.*

The AlphaCoV found in two of five *M. austroriparius* was most similar to ACoV-2-Tb, previously detected in fecal samples from *T. brasiliensis* collected in Argentina in 2017–2018 [76]. In the phylogeny of the concatenated alignment, ACoV-Ma3-FL-2021 formed a well-supported monophyletic subclade with ACoV-2-Tb strains, distinct from classified subgenera. Our results are consistent with prior observation that the ACoV-2-Tb lineage clusters with *Myotis*-associated AlphaCoVs from the USA [76]. Similar detections of closely related AlphaCoVs in geographically distant bat hosts have been reported and may reflect historical cross-species transmission [33]. The relatively long branch leading to our AlphaCoV and its nucleotide divergence from ACoV-2-Tb are consistent with host-associated diversification and/or unsampled regional diversity, raising the possibility of a distinct, *M. austroriparius*-associated lineage. The only other documented AlphaCoV from native Florida bats is a partial RdRp sequence detected in *T. brasiliensis* collected in Gilchrist County in May 2016 [35]. Because our AlphaCoV assembly does not include RdRp, direct comparison with these viruses was not possible. However, RdRp phylogenies place these viruses in a lineage distinct from the AlphaCoV from Argentina closely related to ours, suggesting co-circulation of separate two AlphaCoV lineages in different bat species within Florida [35,76]. In the spike gene phylogeny, ACoV-2-Tb strains clustered in a distant and separate lineage from ACoV-Ma3-FL-2021, while our study sample maintained its close association with *Colacovirus* and *Pedacovirus*. This pattern suggests the ACoV-2-Tb strains may be recombinant in the spike region, which is consistent with Cerri et al.’s finding that ACoV-2-Tb is closely related to the *Colacovirus* subgenus, except for in the spike gene [76]. Our analysis of the spike phylogeny is limited by partial recovery of the spike gene from our sample; therefore future sampling of *M. austroriparius* in North Florida is warranted to better understand the complete spike evolutionary history.

Neither *AlphaCoV* nor *AstV* were detected in samples obtained from *T. brasiliensis* collected across multiple Florida counties. Because RNA viruses are routinely recovered by metagenomic sequencing from bat intestinal/rectal tissues and feces, sample type alone is unlikely to explain this non-detection. This absence may reflect limited sample size and/or species-specific differences in viral shedding dynamics of bat species in north Florida [85]. There is strong evidence in the literature that poor bat health stemming from nutritional stress and/or loss of habitat is associated with increased viral shedding [85,87]. 

Although we cannot determine whether the bats with evidence of AlphaCoV and AstV infection were sick, previous reports of AlphaCoV [88,89,90] and AstV [74,91] in bats did not describe clinical signs of disease, consistent with the asymptomatic presentation commonly observed in reservoir hosts [92,93,94]. Overall, our metagenomic analyses seem to suggest that the bats sampled in north Florida are generally healthy, and while viral detection was limited to individuals roosting in an urban fire station, with no infections observed in bats from designated rural bat houses, we cannot exclude that this was due to the limited sampling in this study. Urban roosting may expose bats to factors such as habitat disturbance, noise, light pollution, and altered food availability, all of which may modulate host immunity, impair health status, and influence viral shedding [34,95,96,97]. Therefore, we should investigate further the hypothesis that this pattern may reflect anthropogenic stressors associated with urban, high-human activity environments compared to more natural settings. Maintaining the health of Florida’s native bat populations is important both for conservation and for mitigating viral shedding-related risks. Expanded, longitudinal sampling of co-roosting species in urban and natural environments, complemented by targeted assays, will be necessary to clarify whether AlphaCoV or AstV transmission occurs between species in north Florida.

The mammalian gut microbiome modulates host susceptibility to infection via effects on physiology, immunity, nutrition, and behavior [10,11,98]. In bats, microbial composition and diversity vary among species, likely reflecting differences in diet, habitat, and seasonal behavior [10,13,18,25,99]. Emerging evidence indicates that gut microbes can modulate antiviral responses, which may contribute to bats’ notable tolerance to viral pathogens [12,13]. Although several studies characterized gut microbiota in members of the genus *Myotis*, this is, to our knowledge, the first investigation of the gut microbial community in *M. austroriparius* and the first microbiome study of bats from Florida. Across bat species, *Corynebacterium* is generally regarded as a commensal genus, [10,100,101,102,103,104,105] so the absence of *Corynebacterium* in the co-infected individual (Ma_Frozen_4) suggests a disruption of the gut microbiome. In this individual, *Staphylococcus* and *Mycoplasma* emerged as notable taxa. Both genera have been reported to display negative associations with other gut bacteria, implying competitive interactions and microbial imbalance under conditions of viral co-infection [106]. Additional indication of altered gut microbiota comes from the presence of *Acinetobacter iwoffi*, which is an opportunistic human pathogen associated with gastroenteritis [107,108,109] and may be in bats as well. This individual exhibited the greatest within-sample (alpha) diversity and a phylogenetically distinct community composition relative to AlphaCoV-only and virus-negative conspecifics. This pattern could reflect a potential synergistic effect of co-infection on microbiome structure, or alternatively, with AstV—an enteric virus—being the primary driver of community shifts [110,111,112,113,114]. Although *AstV* infections are generally considered asymptomatic in bats, they may nonetheless perturb gut communities [115,116]. Wasimuddin et al. reported age-dependent effects in *Artibeus jamaicensis*, with infected adults showing increased diversity and enrichment of potentially pathogenic genera such as *Mycoplasma* and *Acinetobacter* [116]. Consistent with this pattern, the AstV co-infected *M. austroriparius* displayed higher alpha diversity and greater between-sample dissimilarity (Bray–Curtis) than the AlphaCoV-only and virus-negative individuals, along with increased relative abundances of *Mycoplasma*, *Staphylococcus*, and *Acinetobacter*. While these preliminary data are consistent with possible effects of viral co-infection on microbiome structure, they are based on a single individual and therefore warrant further sampling to test the effects of co-infection on gut microbial shifts in *M. austroriparius.* The divergence in microbial community structure observed in one of the healthy individuals (Ma_Frozen_5) is unlikely to be virus-associated as no viral sequences were detected from this specimen. Instead, the predominance of *Mycoplasma* together with reduced alpha diversity suggests a potential enteric *Mycoplasma* infection, consistent with previous associations between decreased diversity and diseased states [106]. Alternatively, we cannot exclude that variation in decomposition time or sample handling prior to deposition at the FLMNH may have contributed to the observed pattern. The causal directionality between viral infection and microbiome state remains unresolved; viruses may reshape gut communities, or particular community configurations may predispose bats to infection [11].

Beyond crop protection, bat predation contributes a clear public-health value in mosquito control [17,117]. Detection of mosquito-associated viruses in bat feces can provide insight into the breadth of mosquito consumption across bat species and habitats. Here, we report the first detection of insect virus HVLV2 in guano of Floridian *T. brasiliensis* likely reflecting dietary intake of mosquitoes, as insectivorous bat fecal viromes are often dominated by diet-derived insect viruses [31]. HVLV2 has been detected in several mNGS studies of multiple *Culex* species and may therefore serve as a useful marker of *Culex* consumption when identified in bat feces [118,119,120,121]. In our study, the clustering of HVLV2 with viruses detected in arbovirus vectors of public health importance, *Cx. erythrothorax* and *Cx. tarsalis* [122,123,124], warrants further investigation to assess the potential role of bat predation in suppressing endemic arbovirus vector populations [40,117,125]. This finding is interesting for several reasons. First, it suggests that Florida bats are predators of *Culex* mosquitoes, the globally widespread vector for several human pathogens, including West Nile virus, St. Louis encephalitis virus, and Japanese encephalitis virus [126]. Second, neither of these species has been reported in Florida; therefore, this finding highlights the importance of studying insect-virus profiles in bat feces, as such data may provide early indicators of geographic expansion by non-native mosquito species. Last, it could suggest association of this virus with other *Culex* species present in north Florida such as *Cx. quinquefasciatus* and *Cx. nigripalpus*, both epidemiologically and ecologically important mosquito species [123,127,128]. Detecting *Culex*-associated viral sequences (such as HVLV2) in bat feces may therefore not only reflect dietary exposure but also provide sentinel evidence of local vector activity and virus circulation within overlapping ecological niches.

Although mammalian viruses often dominate zoonotic discussions, insect-associated viruses captured via mNGS illuminate predator–prey interactions and ecological drivers shaping bat viromes [30]. From a conservation perspective, diet monitoring informs population health, potential shedding risks linked to nutritional stress, and the impacts of anthropogenic disturbance [79,129]. Manual inspection of guano can underestimate mosquito consumption due to the difficulty of identifying digested remains [40,130]. In contrast, passive guano collection coupled with mNGS offers a sensitive, convenient, and cost-effective approach that avoids trapping individuals and circumvents morphological identification [131]. Accordingly, fecal-virome mNGS can provide actionable insights into insect-associated viruses of agricultural and public-health concern and position bats as sentinels for the circulation of insect-borne viruses [132].

This study is constrained by a small sample size and a cross-sectional design, owing to the opportunistic nature of sample acquisition, which limits statistical power, generalizability, and causal inference regarding links between viral detection and microbiome composition. We did not include extraction blanks or other negative controls; thus, prevalence-based contaminant removal (e.g., *decontam* in R) was not possible, and potential low-biomass contaminants cannot be distinguished from true community members. In addition, each group comprised a single sample without biological replication, precluding statistical comparisons (e.g., Kruskal–Wallis, PERMANOVA, differential abundance tests). Accordingly, taxonomic and diversity results are reported descriptively and should be interpreted as exploratory. Future studies will include appropriate controls and replicates to enable formal decontamination and inference. The use of archived museum specimens introduces heterogeneity in preservation history and RNA integrity, and host metadata (e.g., age, sex, reproductive status, diet) were incomplete, leaving potential confounders unaccounted for. Metagenomic assemblies generally resulting in partial genomes and read-based detection cannot always distinguish transient dietary/environmental exposure from active infection (as considered for HVLV2). A general lack of existing data from Florida bats restricts comparison between the data presented here and other studies.

Despite these constraints, our data provide a proof-of-concept for future viral surveillance studies in bat populations in Florida. We report the first detection of HVLV2 in Florida’s T. brasiliensis, the first evidence of *AstV* and *AlphaCoV* co-infection in Florida’s *M. austroriparius*, along with coherent shifts in gut microbial community structure in the co-infected bat, and the first occurrence of *AstV* in Florida bats overall. Our results validate a museum-enabled, integrated virome–microbiome approach and motivate larger, longitudinal sampling with targeted assays to confirm infection status and dissect mechanistic links between viral presence, microbiome state, and host ecology. We highlight the value of using museum specimens as an ethical and convenient resource for investigating the bat virome and microbiome without the need to sacrifice live animals [133]. This approach allows for a comparison of historical trends in virus circulation when compared to newly collected wildlife samples. Our aim is not to bring negative attention to Florida’s bat population, but rather to highlight the worthiness of viral surveillance from a conservation and public health standpoint.

## Figures and Tables

**Table 1:** Description of study bat samples.

Sample Type	Bat Species (N)	Collection Year	Collection County
Feces	*T. brasiliensis* (12)	2024	Alachua
	*T. brasiliensis* (14)	2022	Clay
Anal Swab	*T. brasiliensis* (14)	2022	Clay
Museum Specimen	*M. austroriparius* (5)	2021	Gilchrist
	*T. brasiliensis* (5)	2021	Escambia, Gilchrist, Volusia

**Table 2:** Results of BLAST search of viral contigs.

Virus	Bat Species (Bat ID)	Contig (# bp)	Mean Coverage Depth	BLAST Best Match (Accession)	Percent Identity	Genome Region
AlphaCoV	*M. austroriparius* (Ma_Frozen_3)	k119_6311 (925)	21	ACoV-2-Tb (OP700657.1)	81	5′, ORF1a
		k119_670 (5309)	178	ACoV-2-Tb (OP700657.1)	81	S, ORF3, E, M, N, ORF7, ORF8, 3′
	*M. austroriparius* (Ma_Frozen_4)	k119_1119 (1327)	12	ACoV-2-Tb (OP700657.1)	84	N, ORF7, ORF8, 3′
AstV	*M. austroriparius* (Ma_Frozen_4)	k119_11258 (3292)	209	Bat AstV (MZ218054.1)	80	ORF1b, ORF2
HVLV2	*T. brasiliensis* (Tb_Guano_15)	k141_4344 (9829)	194	HVLV2 (MW434995.1)	99	Polyprotein

## Data Availability

The metagenomic and 16S sequence reads have been deposited at NCBI Sequence Read Archive (SRA) under BioProject identifier (ID) PRJNA1305506. The contigs have been deposited to GenBank under accessions listed in Table 2. Data and code repository available at: https://github.com/paolij/FL_Bat_Virome_Microbiome (accessed on 12 November 2025).

## Supplementary Materials

The following supporting information can be downloaded at: https://www.mdpi.com/article/10.3390/microorganisms13112625/s1. Figure S1: Genome scheme of AstV contig; Figure S2: Likelihood mapping analysis results; Figure S3: Maximum likelihood tree of *AstV* RdRp gene; Figure S4 Maximum likelihood tree of *AstV* ORF2 gene; Figure S5: Genome scheme of AlphaCoV contigs; Figure S6: Maximum likelihood trees of *AlphaCoV* spike, envelope, membrane, and nucleocapsid genes; Figure S7: Genome scheme of HVLV2 contig; Figure S8: Microbial composition of Ma_Frozen_4 and Ma_Frozen_5 at the genus level, comparing sample preparations; Figure S9: Microbial composition of Ma_Frozen_4 and Ma_Frozen_5 at the species level, comparing sample preparations; Figure S10: Shannon alpha diversity comparison of Ma_Frozen_4 and Ma_Frozen_5 sample preparations; Figure S11: Bray–Curtis PCoA of Ma_Frozen_4 and Ma_Frozen_5 comparing secondary-prepared and concentrated samples; Figure S12: Shannon alpha diversity indices for Ma_Frozen samples; Figure S13: Bray–Curtis PCoA of Ma_Frozen samples; Figure S14: PCoA of Ma_Frozen samples based on Weighted UniFrac distances; Figure S15: Species-level microbial community composition of Ma_Frozen samples. Table S1: Complete description of bats sampled in this study; Table S2: Complete description of contigs obtained in this study; Table S3: *AstV* accessions; Table S4: Comparative Genetic Identity of AstV-Ma4-FL-2021; Table S5: *AlphaCoV* accessions; Table S6: Comparative Genetic Identity of *AlphaCoV*; Table S7: *HVLV2* accessions; Table S8: Comparative Genetic Identity of HVLV2.

## Abbreviations

The following abbreviations are used in this manuscript:

AlphaCoV	Alphacoronavirus
AstV	Astrovirus
HVLV2	Hubei virga-like virus 2
mNGS	Metagenomic next-generation sequencing
RdRp	RNA-dependent RNA polymerase
FLMNH	Florida Museum of Natural History
FWC	Florida Fish and Wildlife Conservation Commission
ICTV	International Committee on Taxonomy of Viruses
NGS	Next-generation sequencing
ACoV-2-Tb	Tadarida brasiliensis bat alphacoronavirus 2
