# Supplementary material for: Virome and Microbiome of Florida Bats Illuminate Viral Co-Infections, Dietary Viral Signals, and Gut Microbiome Shifts
Source: Microorganisms. 2025 Nov 19;13(11):2625. doi: 10.3390/microorganisms13112625 (PMC12655238; doi:10.3390/microorganisms13112625)
Supplement: Supplementary file 1 [file microorganisms-13-02625-s001.zip › Supplementary_Figures.pdf]

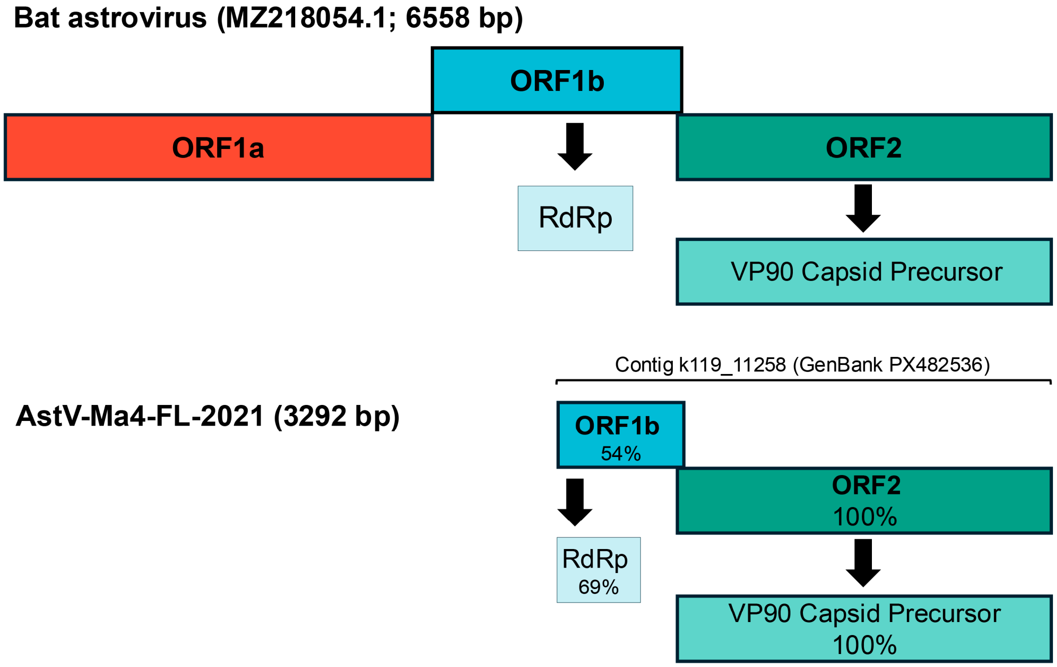

Figure S1. Schematic of the genome coverage for AstV-Ma4-FL-2021 from frozen *M. austroriparius* whole specimen compared to reference Bat astrovirus strain BtAstV/21164-6-B/M.dau/DK/2015 (MZ218054.1). AstV-Ma4-FL-2021 is composed of one contig of 3,292 bp partially covering ORF1b (54%) and fully covering ORF2. Gene products of ORF1b and ORF2 are shown with AstV-Ma4-FL-2021 coverage for RdRp gene (69%) and the VP90 capsid protein precursor (100%).

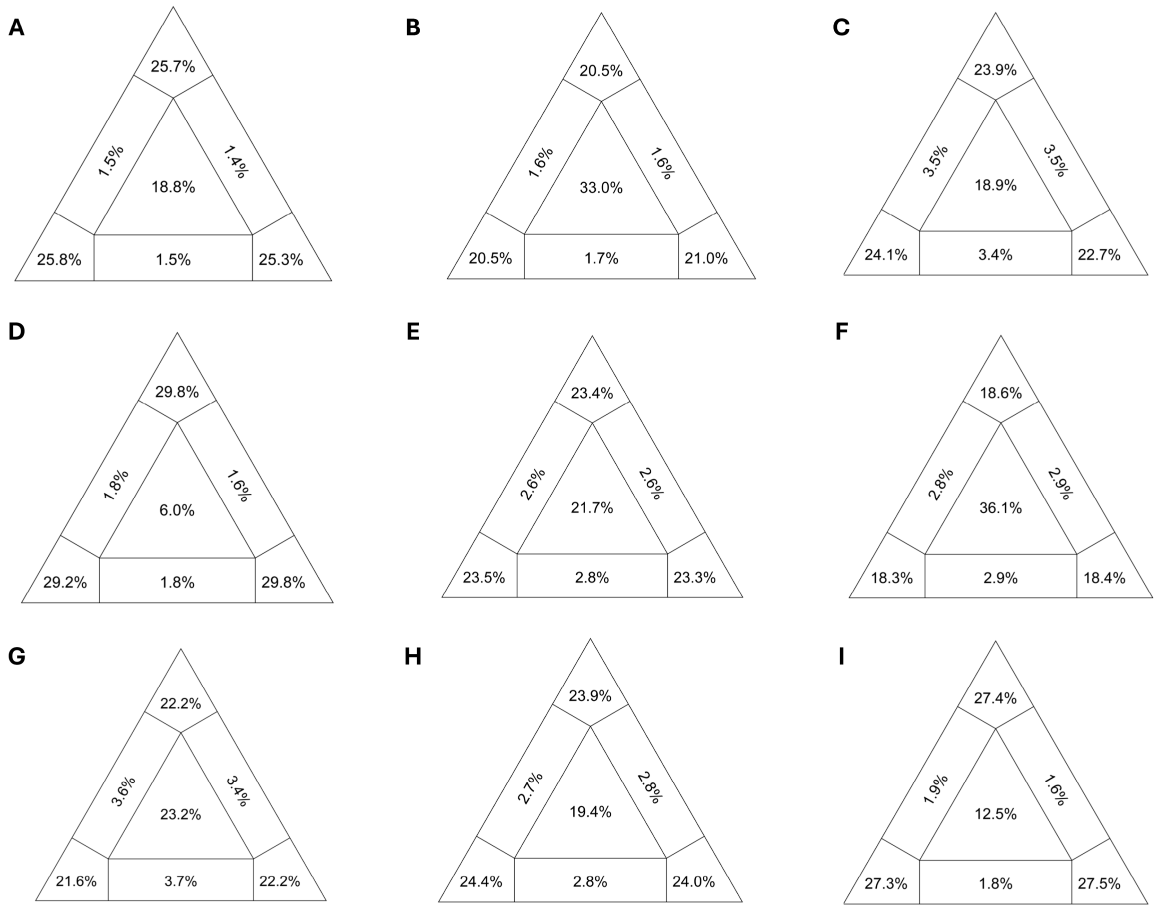

Figure S2. Results of likelihood mapping analysis (LMAP) for all phylogenies. Evidence of robust phylogenetic signal of sequence data indicated by side/center areas of the likelihood mapping including <40% of the unresolved quartets. The maps represent the phylogenies as such: in panel A) AstV-Ma4-FL-2021 full contig; B) AstV-Ma4-FL-2021 ORF2; C) AstV-Ma4-FL-2021 RdRo; D) AlphaCoV-Ma3-FL-2021 concatenated alignment; E) AlphaCoV-Ma3-FL-2021 spike: F) AlphaCoV-Ma3-FL-2021 envelope; G) AlphaCoV-Ma3-FL-2021 membrane; H) AlphaCoV-Ma3-FL-2021 nucleocapsid; and I) HVLV2-Tb15-FL-2024 full contig.

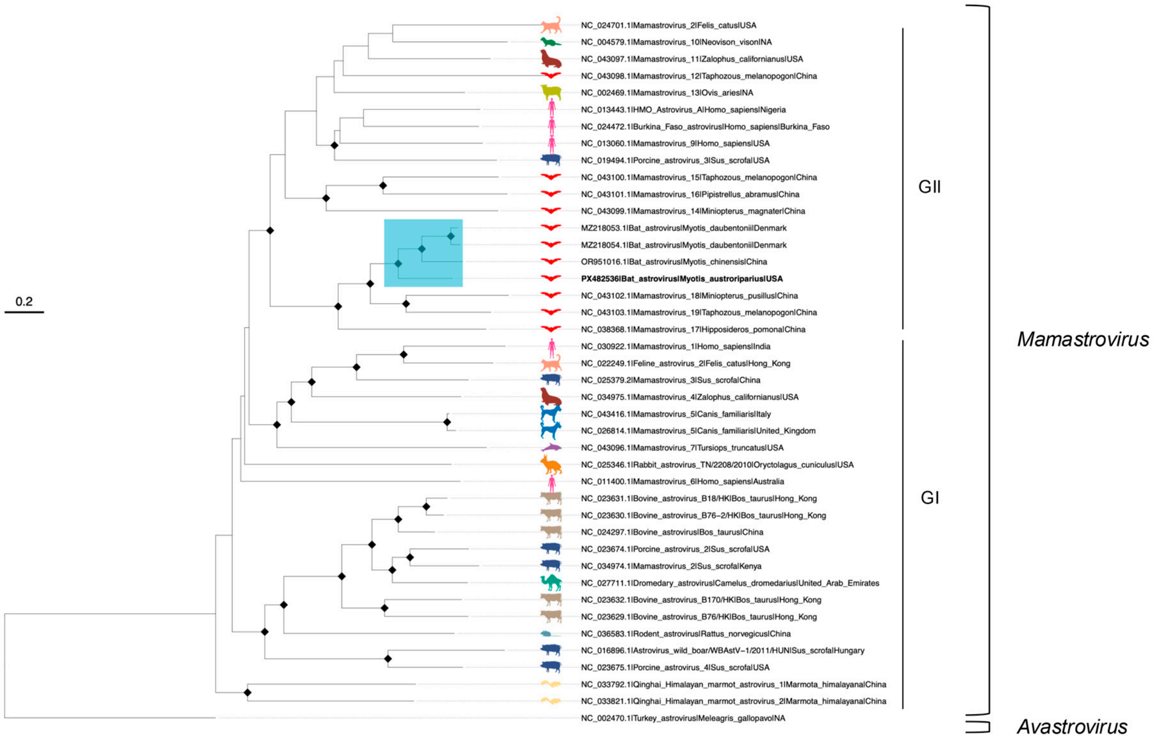

Figure S3. ML tree of AstV found in *M. austroriparius* sampled in Gilchrist County, FL in 2021 (specimen Ma_Frozen_4). The tree is based on the ORF2 alignment of *Mamastrovirus* genomes with the study sample (GTR+F+R5). *Mamastrovirus* genogroups are labeled as GI and GII. The subclade containing AstV-Ma4-FL-2021 (GenBank PX482536), indicated in bold, is highlighted in blue. Sequence hosts are depicted and colored by host species, with bats highlighted in red. Diamonds at nodes indicate ultrafast bootstrap values above 90. Viruses labelled by GenBank accession number, name, and host. Scale bar in nucleotide substitutions per site. Outgroup rooted by *Avastrovirus* Turkey Astrovirus (NC_002480.1).

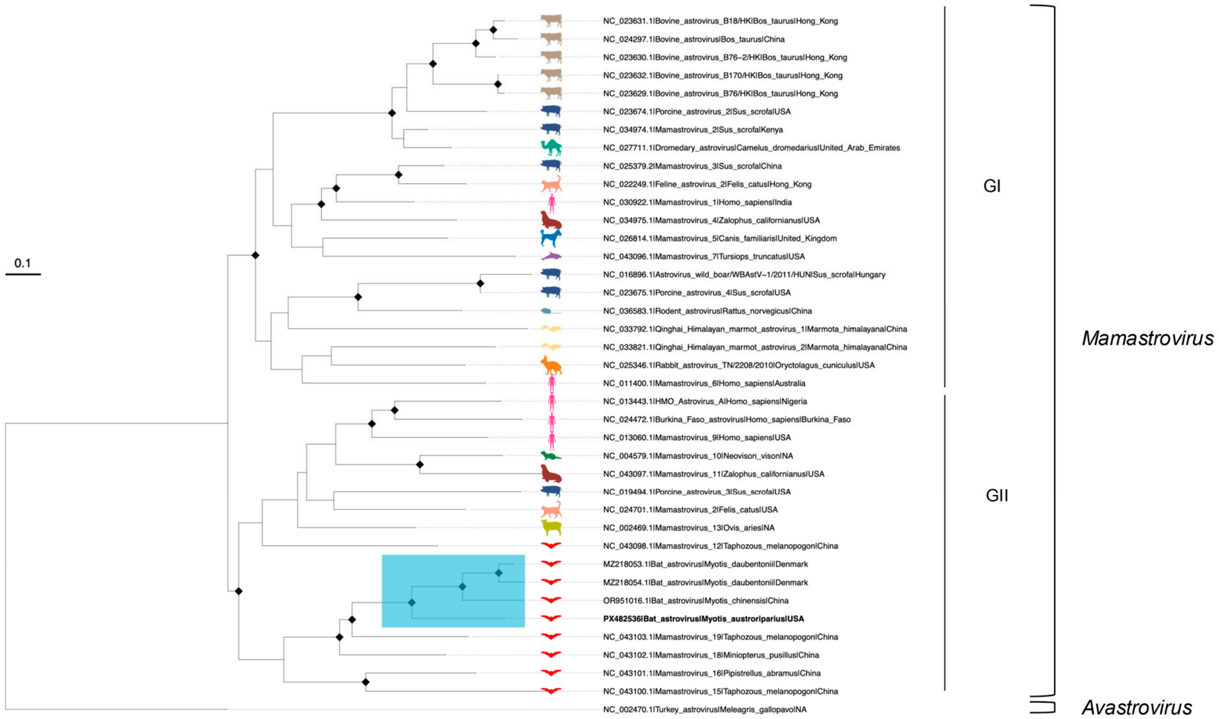

Figure S4. ML tree of AstV found in *M. austroriparius* sampled in Gilchrist County, FL in 2021 (specimen Ma_Frozen_4). The tree is based on the RdRp alignment of *Mamastrovirus* genomes with the study sample (GTR+F+R5). *Mamastrovirus* genogroups are labeled as GI and GII. The subclade containing AstV-Ma4-FL-2021 (GenBank PX482536), indicated in bold, is highlighted in blue. Sequence hosts are depicted and colored by host species, with bats highlighted in red. Diamonds at nodes indicate ultrafast bootstrap values above 90. Viruses labelled by GenBank accession number, name, and host. Scale bar in nucleotide substitutions per site. Outgroup rooted by *Avastrovirus* Turkey Astrovirus (NC_002480.1).

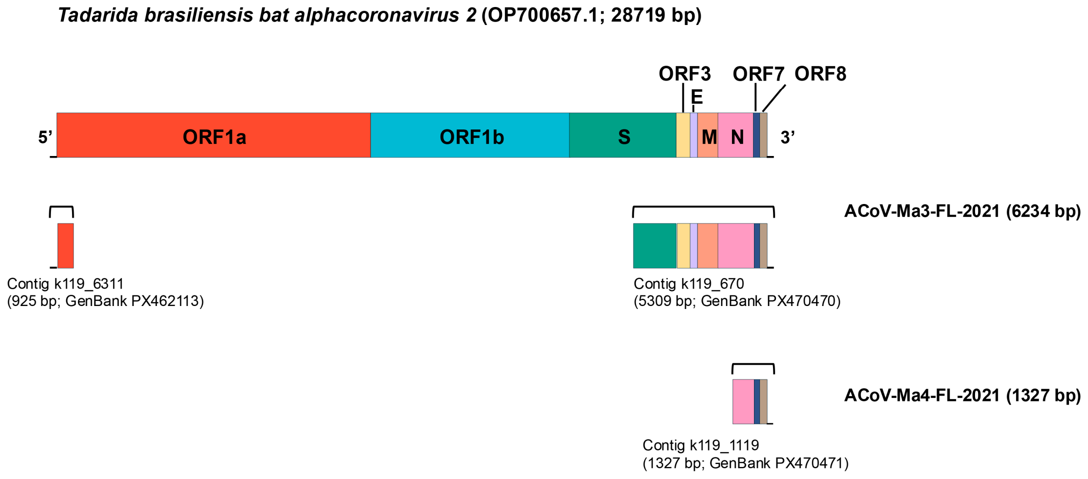

Figure S5. Schematic of AlphaCoV genome coverage for AlphaCoV-Ma3-FL-2021 and AlphaCoV-Ma4-FL-2021 from frozen *M. austroriparius* whole specimens compared to reference virus *Tadarida brasiliensis bat alphacoronavirus 2*. AlphaCoV-Ma3-FL-2021 is comprised of two contigs concatenated together: one contig of 925 bp (k119_6311) covering the 5′ and partially covering ORF1a, and one contig of 5,309 bp (k119_670) partially covering spike gene, and fully covering ORF3, envelope, membrane, nucleocapsid, ORF7, ORF8, and 3′ genomic regions. AlphaCoV-Ma4-FL-2021 is one contig of 1,327 bp (k119_119) partially covering the nucleocapsid gene and fully covering ORF7, ORF8, and 3′ genomic regions.

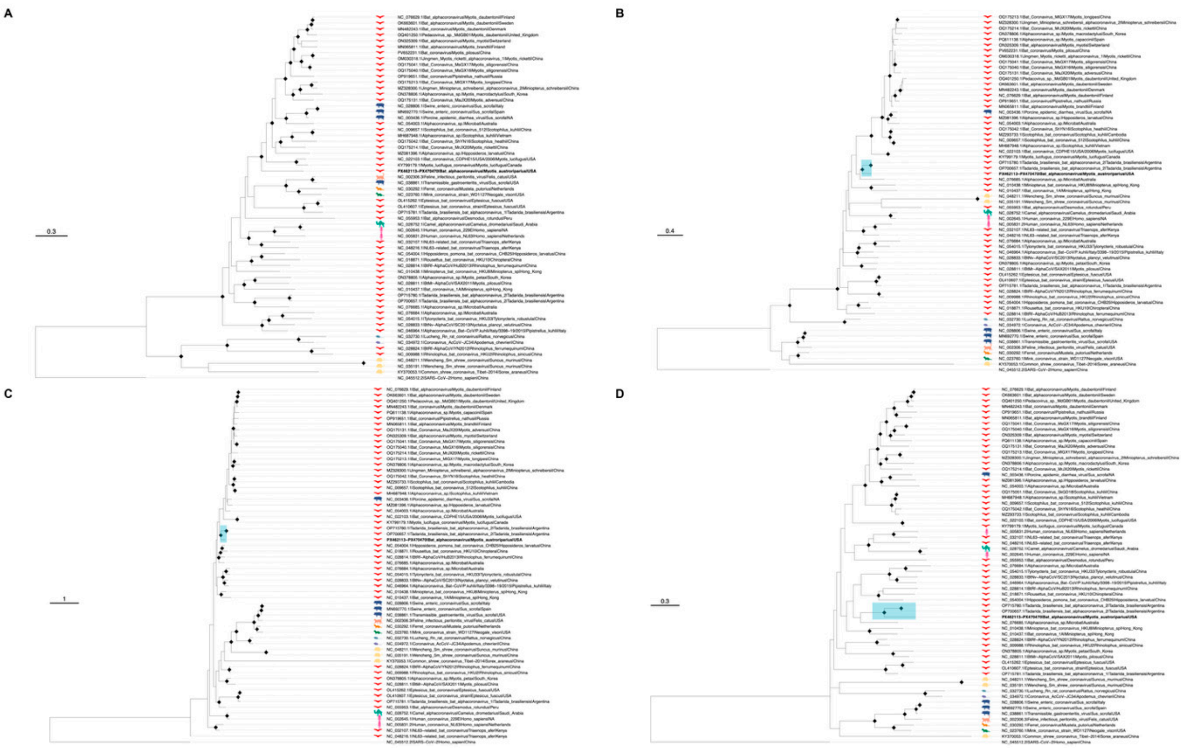

Figure S6. ML trees of AlphaCoV genes found in *M. austroriparius* sampled in Gilchrist County, FL in 2021 (specimen Ma_Frozen_3). The trees in each panel are based on alignments of ACoV-Ma3-FL-2021 with representative AlphaCoVs for the subsequent genes: (**a**) spike (GTR+F+I+R5); (**b**) envelope (TVM+F+I+G4); (**c**) membrane (GTR+F+I+R5); and (**d**) nucleocapsid (GTR+F+R5). The subclade containing ACoV-Ma3-FL-2021, indicated in bold, is highlighted in blue. Sequence hosts are depicted and colored by host species, with bats highlighted in red. Diamonds at nodes indicate ultrafast bootstrap values above 90. Viruses labelled by GenBank accession number, virus name, and host. Scale bar in nucleotide substitutions per site. Outgroup rooted by *Betacoronavirus* SARS-CoV-2 (NC_045512.2).

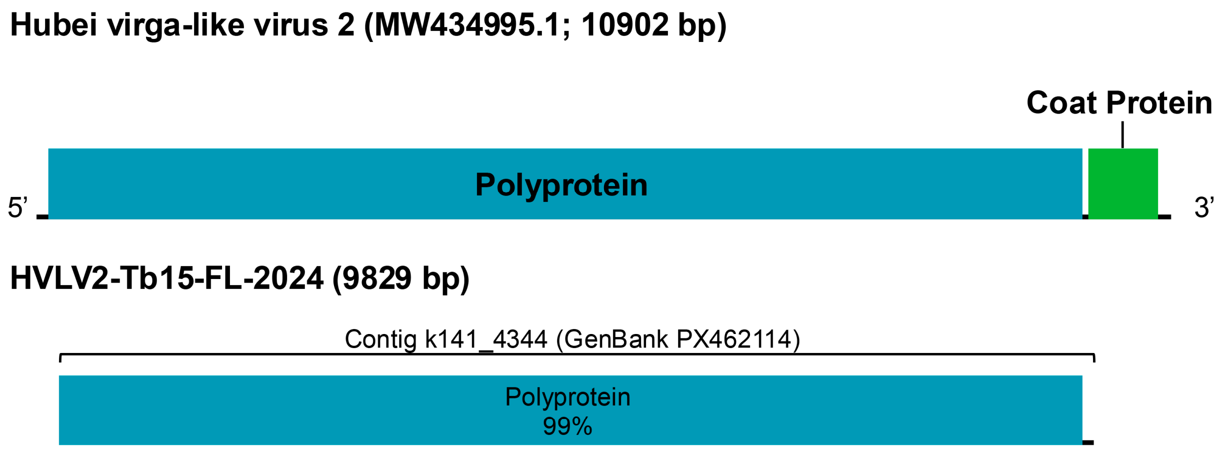

Figure S7. Schematic of HVLV2 genome coverage for HVLV2-Tb15-FL-2024 compared to a reference HVLV2 genome sampled from *Cx. erythrothorax* (MW434995.1). HVLV2-Tb15-FL-2024 is comprised of one contig of 9,829 bp (k141_4344) covering 99% of the polyprotein region and none of the coat protein.

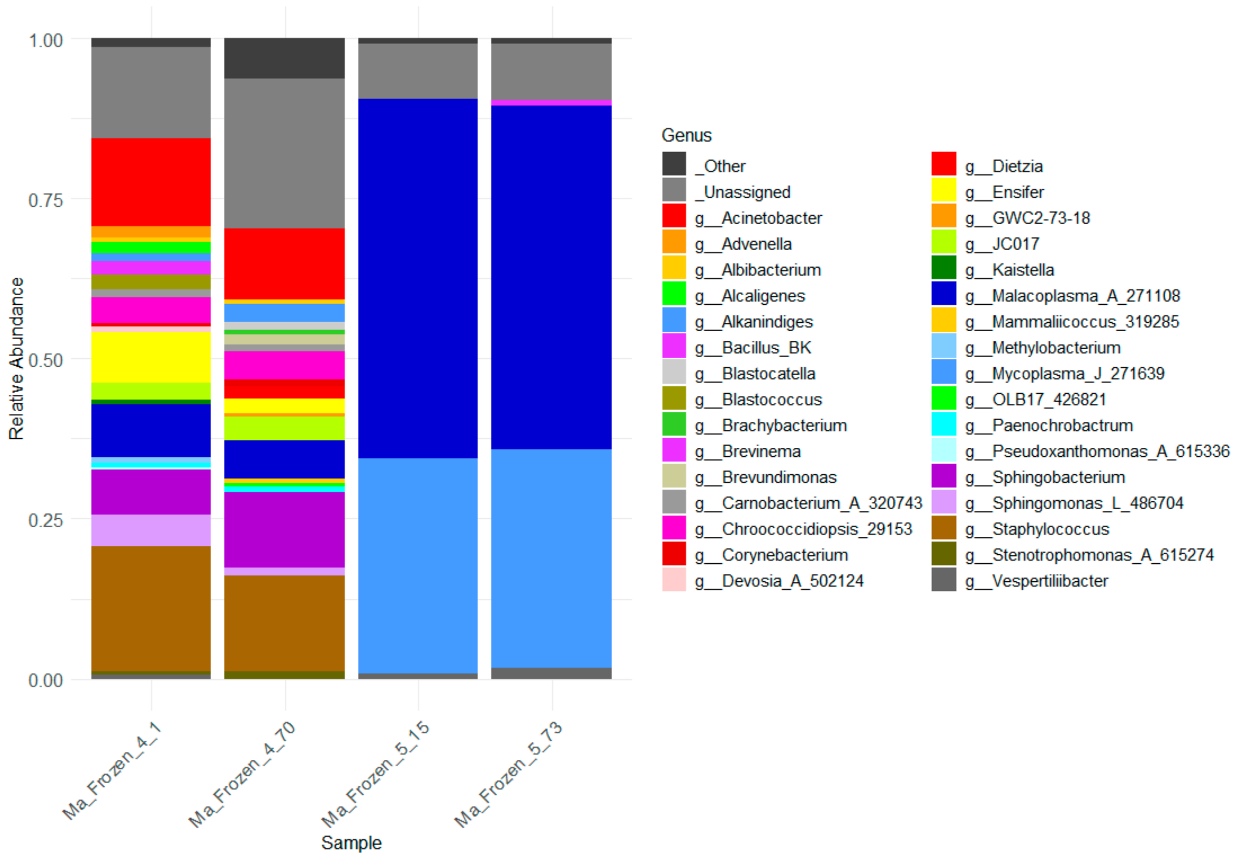

Figure S8. Microbial community composition of Ma_Frozen_4 and Ma_Frozen_5 at the genus level, comparing secondary-prepared and concentrated samples. Only taxa with ≥0.5% relative abundance are shown. For taxonomic assignment, a Naive Bayes classifier was trained on SILVA 138 reference sequences trimmed in silico to the V3–V4 region using primers 341F (CCTACGGGNGGCWGCAG) and 806R (GACTACHVGGGTATCTAATCC).

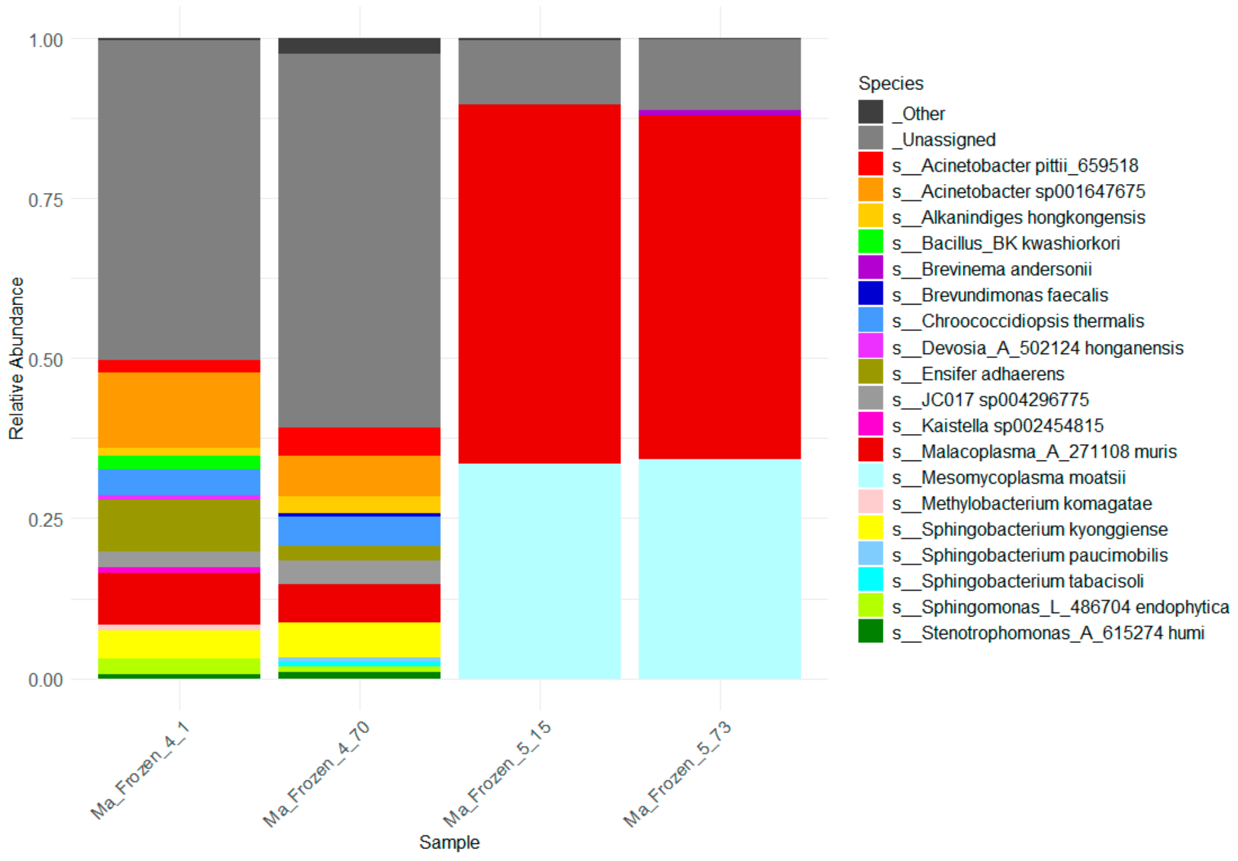

Figure S9. Microbial community composition of Ma_Frozen_4 and Ma_Frozen_5 at the species level, comparing secondary-prepared and concentrated samples. Only taxa with ≥0.5% relative abundance are shown. For taxonomic assignment, a Naive Bayes classifier was trained on SILVA 138 reference sequences trimmed in silico to the V3–V4 region using primers 341F (CCTACGGGNGGCWGCAG) and 806R (GACTACHVGGGTATCTAATCC).

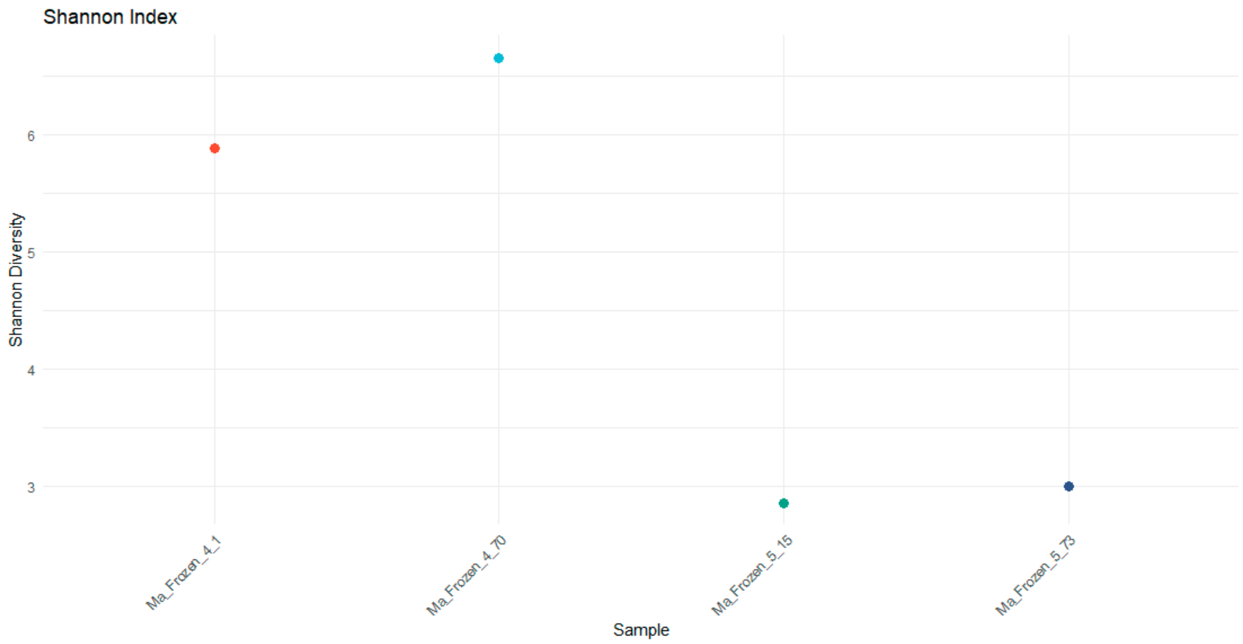

Figure S10. Shannon alpha diversity indices for Ma_Frozen_4 and Ma_Frozen_5, comparing secondary-prepared and concentrated samples. Shannon diversity was assessed with group-level differences were tested with Kruskal–Wallis as implemented in QIIME 2's alpha-group-significance.

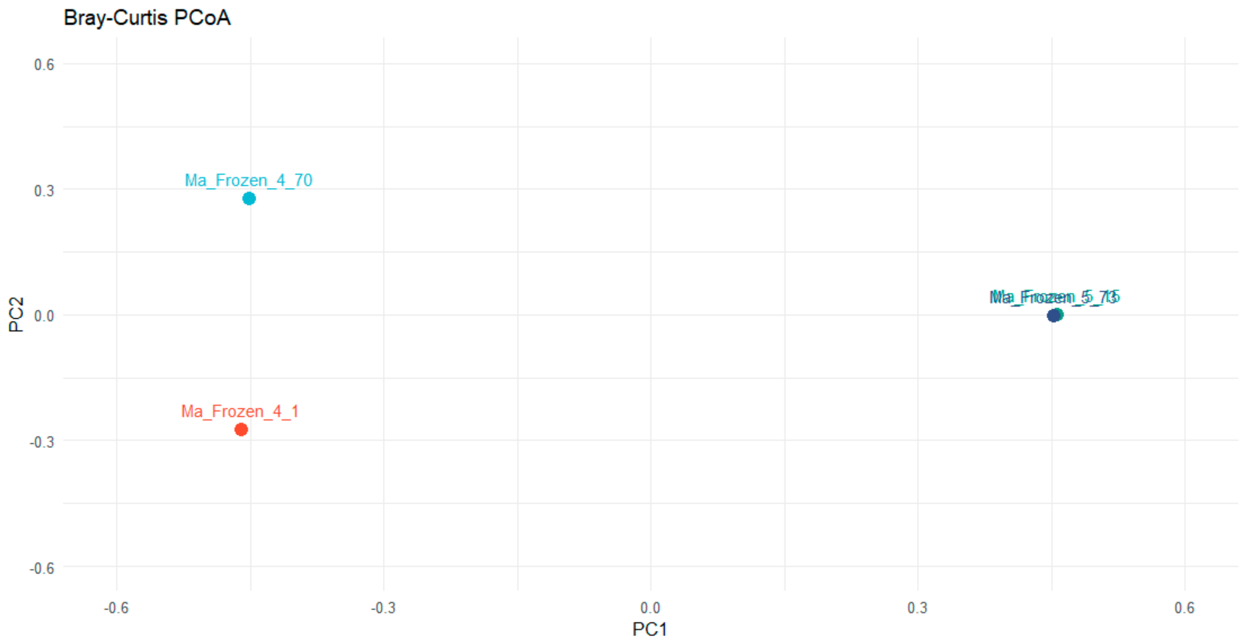

Figure S11. Principal Coordinate Analysis (PCoA) based on Bray–Curtis dissimilarity, comparing secondary-prepared and concentrated samples of Ma_Frozen_4 and Ma_Frozen_5. Differences in community composition were tested by PERMANOVA on the Bray–Curtis distance matrix (999 permutations).

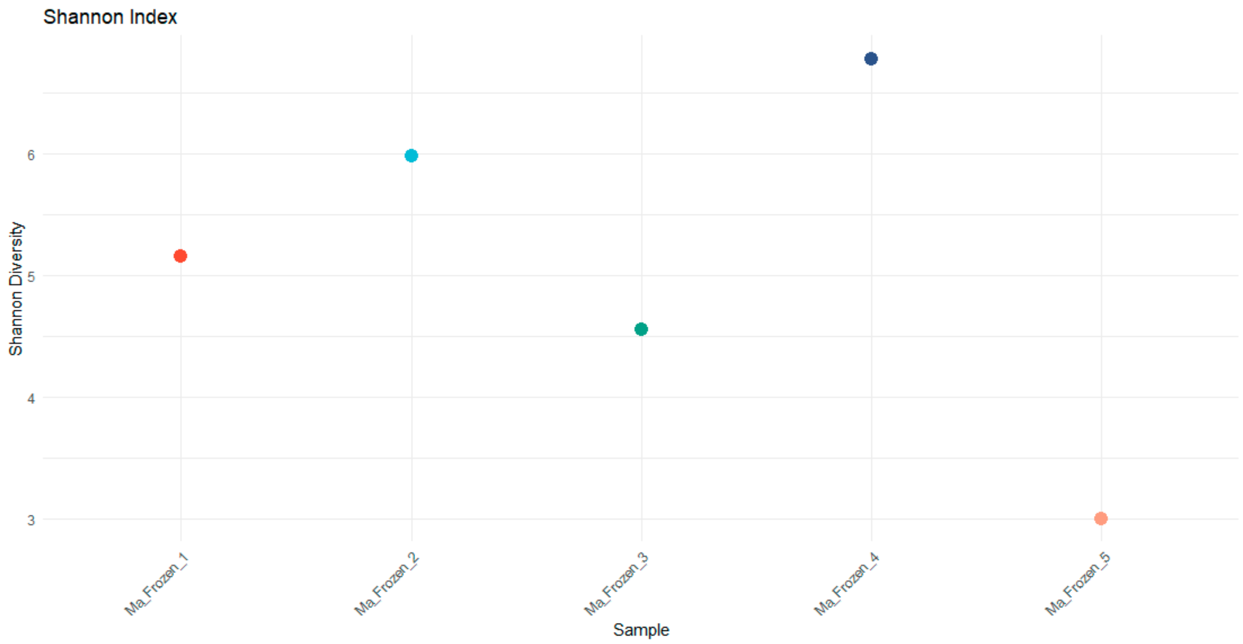

Figure S12. Shannon alpha diversity indices across all frozen *M. austroriparius* samples. Shannon diversity was assessed with group-level differences were tested with Kruskal–Wallis as implemented in QIIME 2's alpha-group-significance.

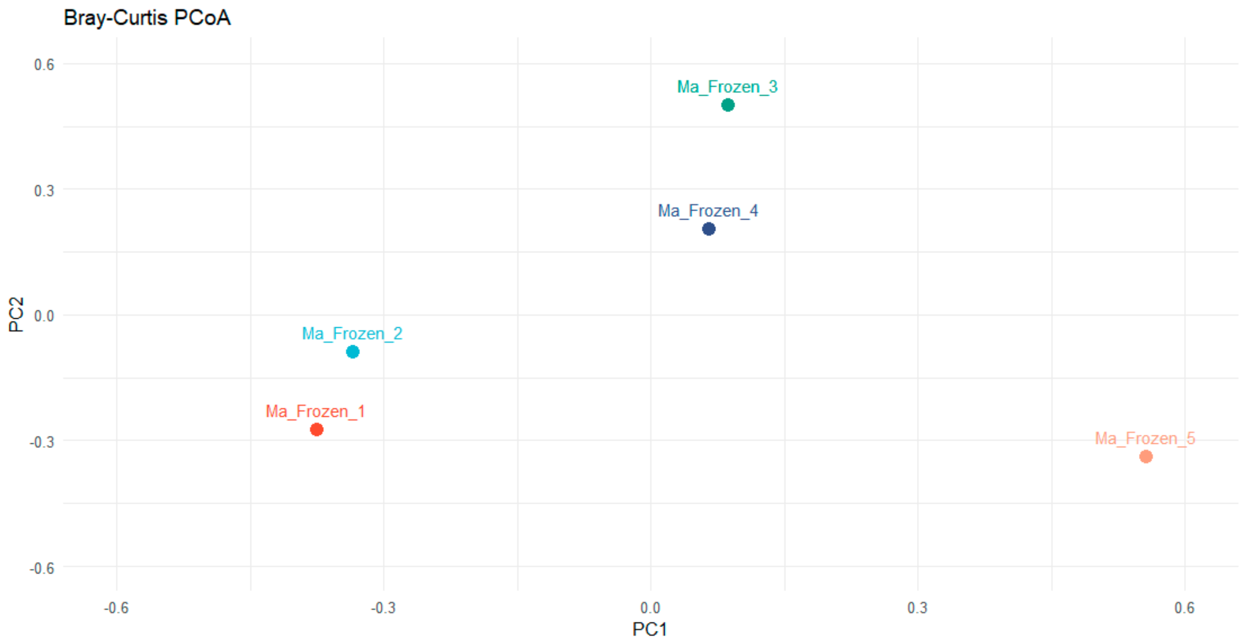

Figure S13. Principal Coordinate Analysis (PCoA) based on Bray–Curtis dissimilarity across all frozen *M. austroriparius* samples. Differences in community composition were tested by PERMANOVA on the Bray–Curtis distance matrix (999 permutations).

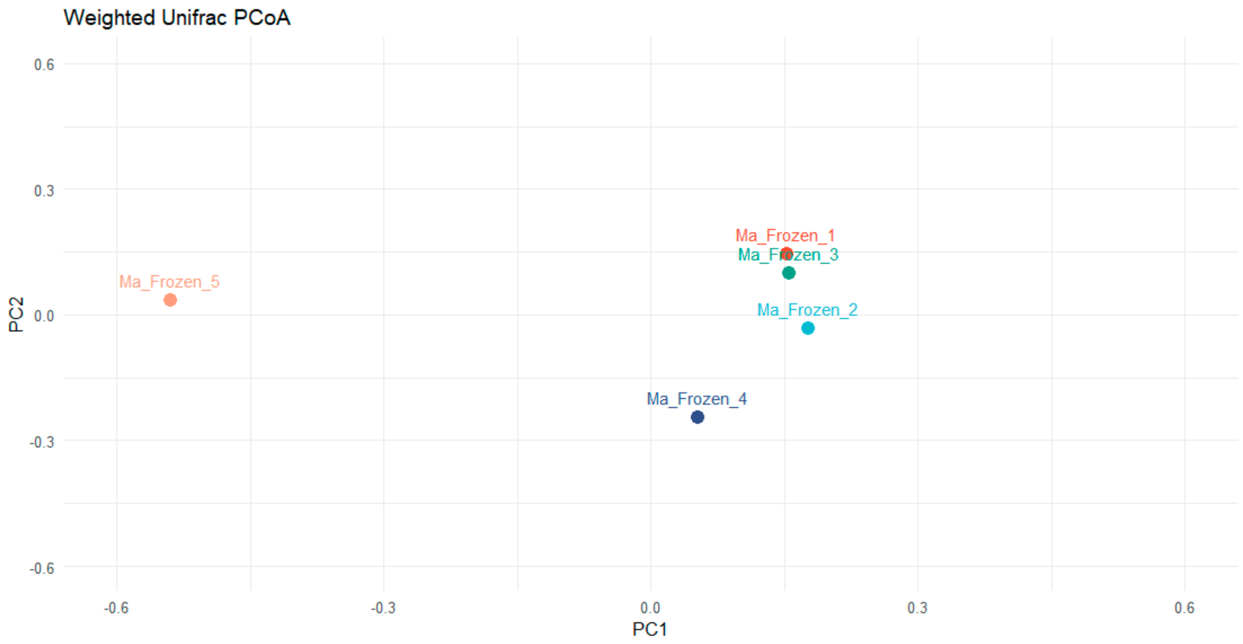

Figure S14. Principal Coordinate Analysis (PCoA) based on Weighted UniFrac distances across all frozen *M. austroriparius* samples. Differences in community composition were tested by PERMANOVA on the Weighted UniFrac distance matrix (999 permutations).

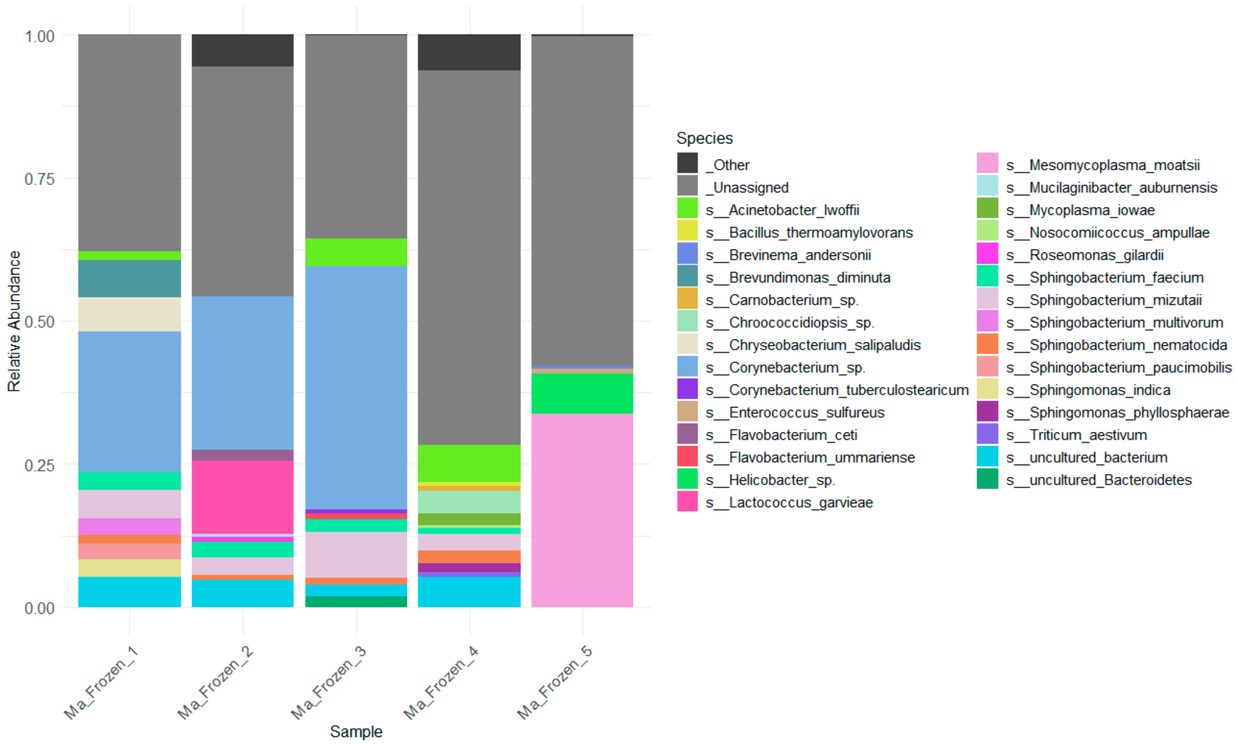

Figure S15. Microbial community composition across all frozen *M. austroriparius* samples at the species level. Only taxa with ≥1% relative abundance are shown. For taxonomic assignment, a Naive Bayes classifier was trained on SILVA 138 reference sequences trimmed in silico to the V3–V4 region using primers 341F (CCTACGGGNGGCWGCAG) and 806R (GACTACHVGGGTATCTAATCC).
